# Exhausting repetitive piano tasks lead to local forearm manifestation of muscle fatigue and negatively affect musical parameters

**DOI:** 10.1038/s41598-021-87403-8

**Published:** 2021-04-14

**Authors:** Etienne Goubault, Felipe Verdugo, Justine Pelletier, Caroline Traube, Mickaël Begon, Fabien Dal Maso

**Affiliations:** 1 Laboratoire de Simulation et Modélisation du Mouvement, École de Kinésiologie et des Sciences de l’activité Physique, Université de Montréal, 1700 Rue Jacques-Tétreault, Laval, QC Canada; 2 Input Devices and Music Interaction Laboratory, Centre for Interdisciplinary Research in Music Media and Technology, Schulich School of Music, McGill University, Montreal, QC Canada; 3 EXPRESSION Team, Université Bretagne-Sud, Vannes, France; 4 Laboratoire Arts vivants et interdisciplinarité, Département de danse, Université du Québec à Montréal, Montreal, QC Canada; 5 Laboratoire de recherche sur le geste musicien, Faculté de musique, Université de Montréal, Montreal, QC Canada; 6 Sainte-Justine Hospital Research Center, Montreal, QC Canada; 7 Centre interdisciplinaire de recherche sur le cerveau et l’apprentissage, Montréal, QC Canada

**Keywords:** Risk factors, Biomarkers

## Abstract

Muscle fatigue is considered as a risk factor for developing playing-related muscular disorders among professional pianists and could affect musical performance. This study investigated in 50 pianists the effect of fatiguing repetitive piano sequences on the development of forearm muscle fatigue and on piano performance parameters. Results showed signs of myoelectric manifestation of fatigue in the 42-electromyographic bipolar electrodes positioned on the forearm to record finger and wrist flexor and extensor muscles, through a significant non-constant decrease of instantaneous median frequency during two repetitive *Digital* (right-hand 16-tones sequence) and *Chord* (right-hand chords sequence) excerpts, with extensor muscles showing greater signs of fatigue than flexor muscles. In addition, muscle fatigue negatively affected key velocity, a central feature of piano sound intensity, in both *Digital* and *Chord* excerpts, and note-events, a fundamental aspect of musicians’ performance parameter, in the *Chord* excerpt only. This result highlights that muscle fatigue may alter differently pianists’ musical performance according to the characteristics of the piece played.

## Introduction

Professional piano performance involves complex motor skills achieved through the repetition of upper-limb fast movements over extended periods of time. Highly repetitive practice combined with long-lasting unnatural static postures make professional musicians at high risk of developing playing-related muscular disorders (PRMDs)^1,2^. PRMDs are the most frequent work-related problems among musicians^3–5^, and can become chronic among pianists, causing severe pain and disability^6^. These disorders mainly involve pianists’ upper body with a higher prevalence reported at the forearm and wrist^7–9^, the lateral epicondylitis^7,8^ being the most common injury. Muscle fatigue caused by the repetition of long-hours of practice is critical in the development of PRMDs^2^.

Electromyography (EMG) is a widely used measure to assess the signs of myoelectric manifestation of fatigue (MMF)^10^. More specifically, the decrease of EMG median frequency is considered as a typical indicator of muscle fatigue^11–13^, due to its linear relationship with the conduction velocity of the active motor units, which decreases with muscle fatigue^10,11^. The median frequency of EMG recordings was shown to be a reliable parameter of MMF during sustained rectus femoris, vastus lateralis and vastus medialis muscles contractions^14^. Additionally, EMG frequency content also shown a fair to good relative reproducibility during sustained contractions at 50% MVC^15^ of extensor forearm muscles and dynamic contractions of arm and forearm muscles^16–18^. Time–frequency analyses recently introduced to investigate instantaneous EMG median frequency^11,19^ have been used to assess signs of MMF during high-^19–21^ and low-load^22^ dynamic tasks. To our knowledge, signs of MMF in pianists has been investigated in only one study^23^, where participants alternated between a five-finger repetitive piano excerpt and a fatiguing task. However, the fatigue task in this study consisted of performing loaded wrist flexion–extensions with the palm facing down, therefore targeting mostly wrist extensor muscles, while piano playing involves moderate to relatively high muscle activation levels of wrist and finger flexors and extensors^7,24,25^. Consequently, although their results showed that signs of MMF occurred mainly in wrist and finger extensor muscles, it is still unclear how muscle fatigue evolves in wrist and finger flexor and extensor muscles during actual piano playing. Interestingly, during repetitive keyboard typing tasks, a low load and dynamical activity like to piano playing, previous studies have shown significant signs of MMF in finger extensors^26–28^. Moreover, signs of MMF is not homogeneous within a given muscle but region-specific^29,30^. Indeed, the decrease of median frequency differs between EMG channels arranged on a muscle according to an electrode matrix. While traditional bipolar EMG records muscle activity on a small muscle portion, the use of high-density EMG (HD-EMG) increases the spatial sampling information within a single muscle^31–33^ and will improve our understanding of MMF, which could help to better prevent PRMDs in pianists.

Muscle fatigue in musicians is not only a risk factor for the development of PRMDs, it is also detrimental for motor control and therefore musical performance. Indeed, it was suggested that the general musical performance of wind instrumentalists would be affected by muscle fatigue^34^. However, how muscle fatigue affects musical parameters related to piano performance, previously assessed via sound intensity, note-events, and timing between note onsets^35–38^, is still to be determined. Interestingly, during the repetition of movements, muscle fatigue altered the precision of submaximal force output, which can be related to the sound intensity of the notes played^39^. Then, the force output fluctuates around the target force hindering accuracy performance and finally prohibiting effective task performance. Other studies showed that muscle fatigue also affects timing and coordination impeding to successfully reproduce movements^40,41^. Indeed, in a rapid elbow flexion–extension pointing task, triceps fatigue caused an undershoot of the final position during extension^42^, suggesting that note-events and timing can also be affected by muscle fatigue.

The aims of this study were to assess in expert pianists the effect of a fatiguing repetitive *Digital* (right-hand 16-tones sequence) and *Chord* (right-hand chords sequence) piano sequences on (1) the evolution of forearm muscle fatigue, and (2) the piano performance parameters at the end of the piano tasks. We hypothesized that finger and wrist flexor and extensor muscles will show signs of MMF and that finger and wrist extensors will show greater signs of MMF than finger and wrist flexors^7,24,26–28,43^. We also expected that muscle fatigue would have a negative impact on main piano performance parameters as it alters motor control^40–42,44–47^.

## Results

### Group clustering

**Figure 1:**
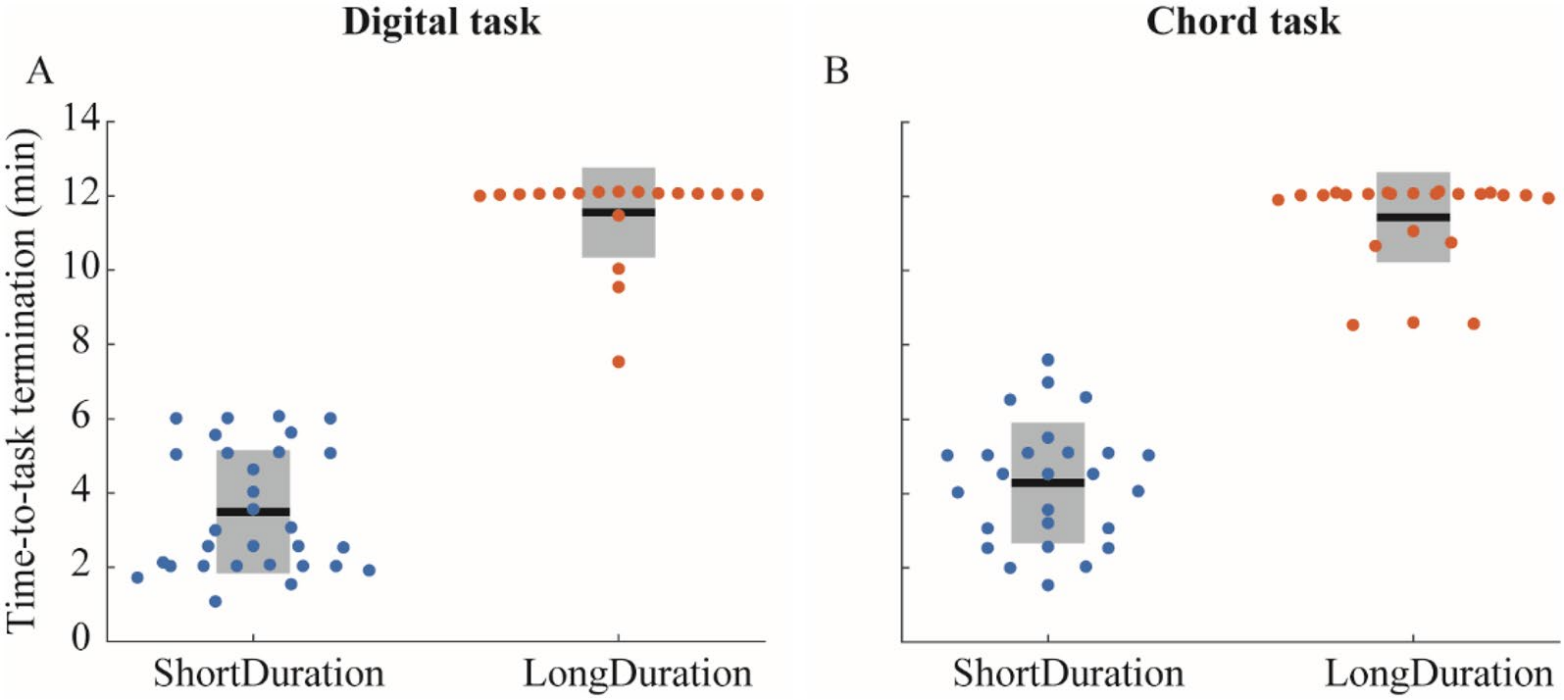
Time-to-task termination for (**A**) the *Digital* task and (**B**) the *Chord* task. Black horizontal lines represent the mean duration for each group, grey boxes represent the standard deviation, and dots represent each participants’ data.

One participant was excluded for both *Digital* and *Chord* tasks because of missing EMG data. In both *Digital* and *Chord* tasks (Appendix-A1), two groups were identified using the k-means clustering method, with a silhouette coefficient of 0.90 and 0.88, respectively. The groups were termed as *ShortDuration* and *LongDuration* groups, and lasted on average 209.7 ± 99.6 s and 693.2 ± 72.7 s for the *Digital* task, respectively, and 257.2 ± 97.4 s and 686.2 ± 72.8 s for the *Chord* task, respectively (Fig. 1). Both groups significantly differed by the time-to-task termination only (Table 1). As can be seen in Fig. 1 showing participants’ individual time-to-task termination, 15 out of the 19 participants and 17 out of the 23 participants of the *LongDuration* group reached 12 min for the *Digital* and *Chord* tasks, respectively. Other stratification parameters such as age, mass, experience, practice time per day, and maximal voluntary contraction (MVC) did not differ between groups (Table 1).

**Table 1:** Demographic data of participants (mean ± SD).

	Digital task			Chord task		
	ShortDuration	LongDuration	Statistical test	ShortDuration	LongDuration	Statistical test
	N = 30	N = 19		N = 26	N = 23	
Left handed	2	5	*X*^2^ (1,49) = 2.24; p = 0.13	5	2	*X*^2^ (1,49) = 0.41; p = 0.52
Sex	9 ♀	10 ♀	*X*^2^ (1,49) = 2.51; p = 0.11	11 ♀	8 ♀	*X*^2^(1,49) = 0.29; p = 0.59
Time to termination (s)	209.7 ± 99.6	693.2 ± 72.7	t(47) = − 19.29; **p < 0.001**	257.2 ± 97.4	686.2 ± 72.8	t(47) = − 17.24; **p = < 0.001**
Age (years)	27.47 ± 8.80	29.84 ± 8.62	t(47) = − 1.24; p = 0.359	27.42 ± 5.08	29.61 ± 7.90	t(47) = − 1.16; p = 0.359
Mass (kg)	66.51 ± 9.98	66.45 ± 13.52	t(47) = 0.24; p = 0.809	65.26 ± 11.42	67.33 ± 13.24	t(47) = − 0.84; p = 0.499
Experience (years)	19.23 ± 6.20	21.97 ± 8.64	t(47) = − 1.29; p = 0.359	19.25 ± 6.50	21.65 ± 7.97	t(47) = − 1.16; p = 0.359
Practice (h/day)*	3.41 ± 1.49	4.08 ± 1.18	t(47) = − 1.66; p = 0.359	3.26 ± 1.49	4.07 ± 1.18	t(47) = − 2.08; p = 0.359
MVC** (N)	319.1 ± 85.3	290.2 ± 75.8	t(47) = 1.20; p = 0.359	304.2 ± 83.3	312.8 ± 82.4	t(47) = − 0.36; p = 0.796

*in the past year. **MVC maximal voluntary contraction. Bold values highlight significant differences between groups (α = 0.05).

### Rate of perceived exertion

**Figure 2:**
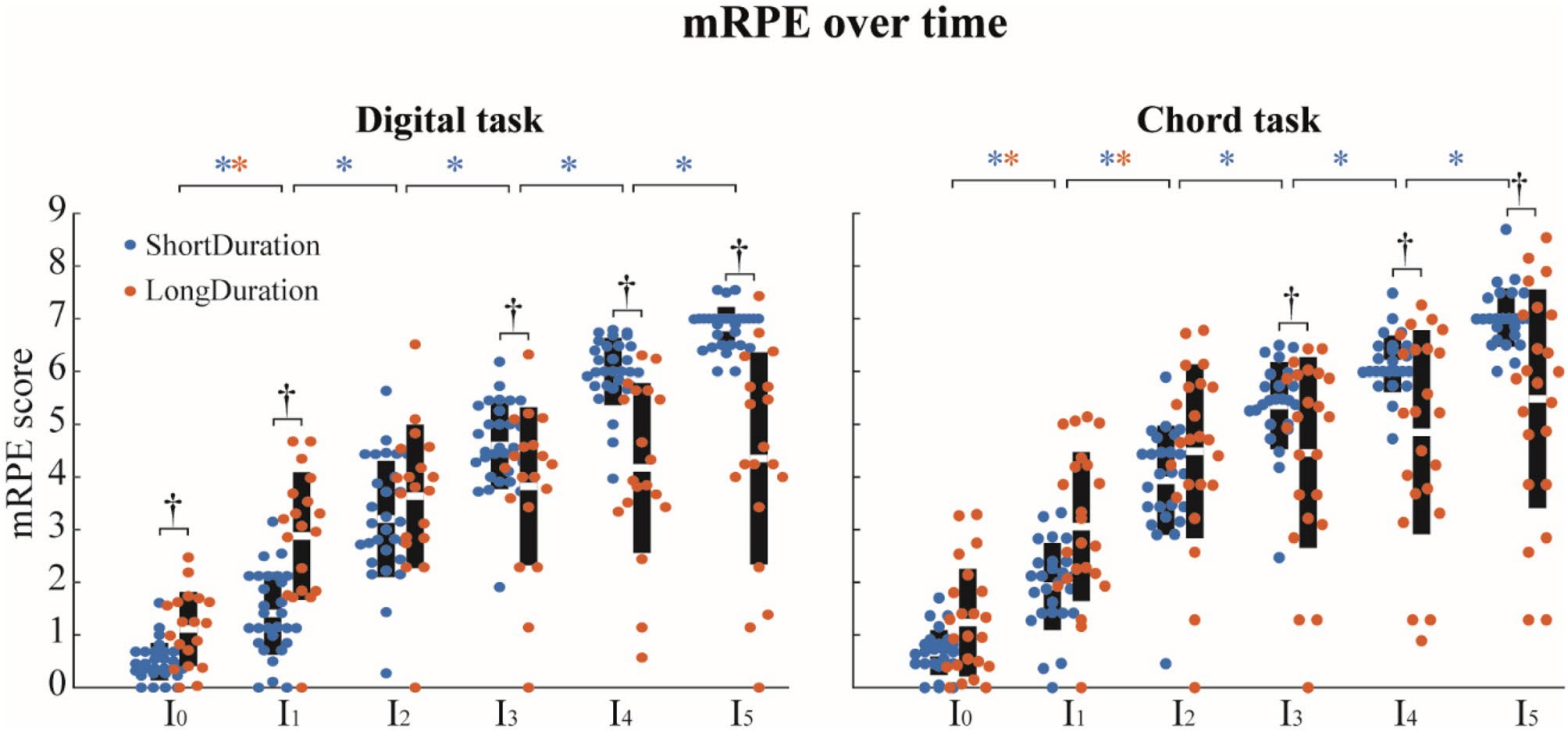
Mean ± standard deviation of mRPE score for each interval for the *Digital* task (left panel) and the *Chord* task (right panel). White horizontal lines represent the mean mRPE score for each group, black boxes represent the standard deviation, and dots represent each participants’ data. Blue and red asterisks represents significant differences between successive intervals for the *ShortDuration* and *LongDuration* group, respectively. ^†^Significant differences between groups (α = 0.05).

In both *Digital* and *Chord* tasks, there was a significant Group–Time interaction (F(5) = 32.67, p < 0.001 and F(5) = 47.36, p < 0.001, respectively) on the modified CR-10 Borg scale (mRPE: modified rate of perceived exertion)^48^. In both tasks, mRPE scores increased for both groups over time, with a greater increase for the *ShortDuration* than the *LongDuration* group (Fig. 2). Tukey post-hoc analysis revealed that the mRPE score increased significantly along the entire tasks for the *ShortDuration* group (ES = 1.38 and ES = 1.26 respectively for the *Digital* and *Chord* task), while the mRPE score increased only until I_1_ for the *Digital* task (ES = 2.38) and until I_2_ for the *Chord* task (ES = 1.05) for the *LongDuration* group. Tukey post-hoc analysis also revealed that the *ShortDuration* group had a lower mRPE score than the *LongDuration* group for I_0_ and I_1_ of the *Digital* task (ES = 0.76). Additionally, the *ShortDuration* group had a higher mRPE score than the *LongDuration* group at the end of both tasks (i.e., I_3_: ES = 1.52, I_4_: ES = 1.76, and I_5_: ES = 1.76) (Fig. 2).

### Evolution of electromyographic median frequency

#### Digital task

**Figure 3:**
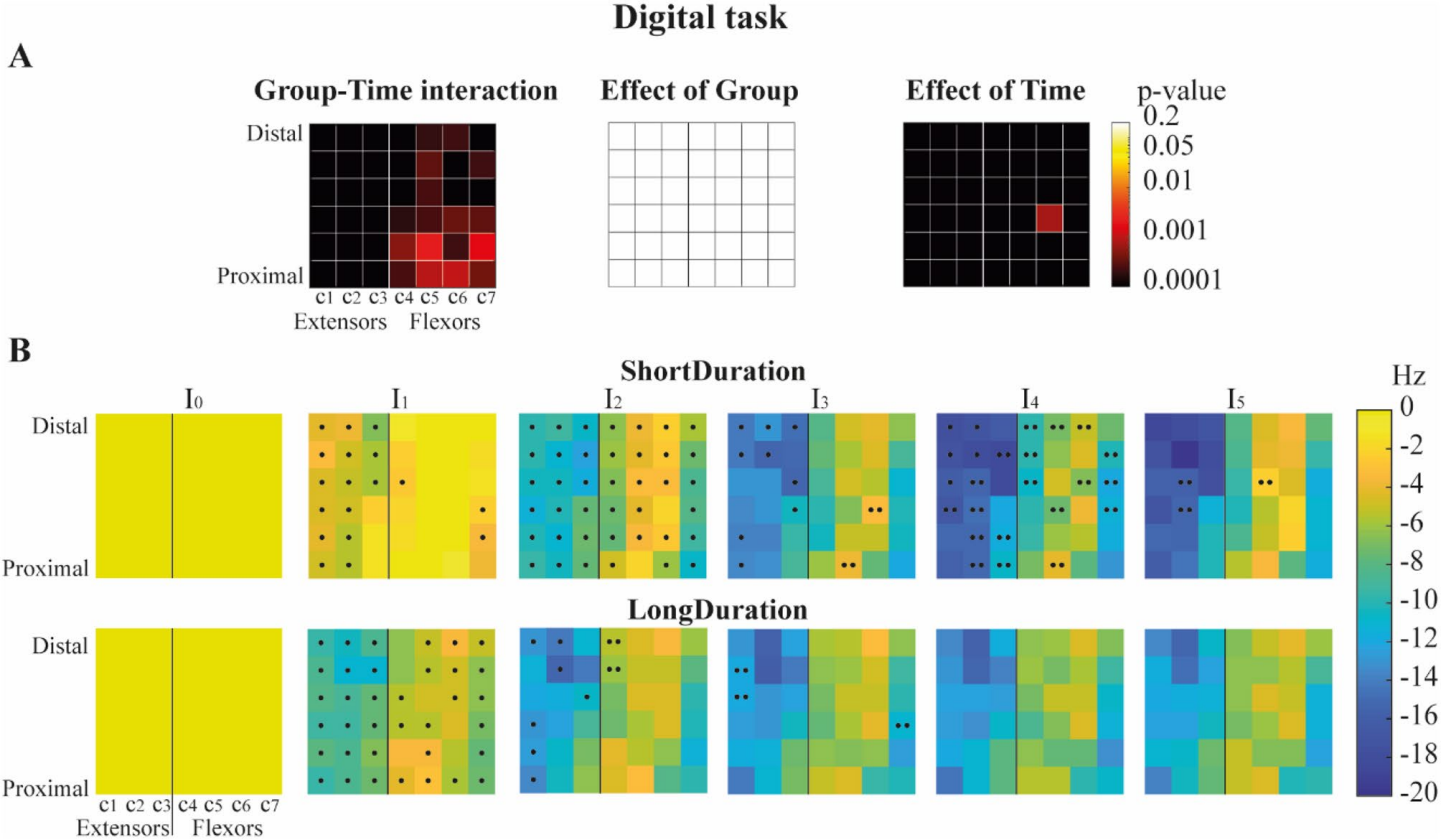
(**A**) colormap representation of ANOVA results’ p values for Group–Time interaction, main effects of Group, and Time for the *Digital* task. Columns of electrodes are labelled *c1* to *c7* as illustrated on the Fig. 8B. (**B**) Average change from baseline (I_0_) representation of the participants’ EMG median frequency of the forearm muscles throughout the duration of the task (I_0:5_), for the *ShortDuration* (superior panel) and *LongDuration* (inferior panel) groups. Note that ANOVA were performed on EMG median frequency values and that variation from baseline was for representation only. Dots indicate significant differences revealed by post-hoc analyses. One dot indicates a significant difference between time intervals I_i_ and I_i−1_ for a given pair of electrode, while two dots indicates a significant difference between time intervals I_i_ and I_i−2_. For example, at time interval I_4_ for the *ShortDuration* group, the dot on the left column (*c1*) of the top row indicates a significant difference between the EMG median frequency at time intervals I_3_ and I_4_, while the two dots on the middle column (*c4*) of the top row indicates a significant difference between the EMG median frequency at time intervals I_2_ and I_4_.

There was a significant Group-Time interaction for the EMG median frequency for all the forearm bipolar signals (Fig. 3A). The median frequency decreased for both groups over time, with a greater decrease in the *ShortDuration* group (ES = 1.06) than the *LongDuration* group (ES = 1.00). Tukey post-hoc analyses revealed that for *ShortDuration* group, median frequency significantly decreased along the task from I_0_ to I_4_ for most of the 42 bipolar signals. For the *LongDuration* group, median frequency significantly decreased between I_0_ and I_1_ for 36 out of 42 bipolar signals and between I_1_ and I_2_ for 7 bipolar signals in the lateral and posterior parts of the forearm, but remained mostly unchanged during the rest of the task (Fig. 3B; Fig. 2 in Appendix-A4).

**Figure 4:**
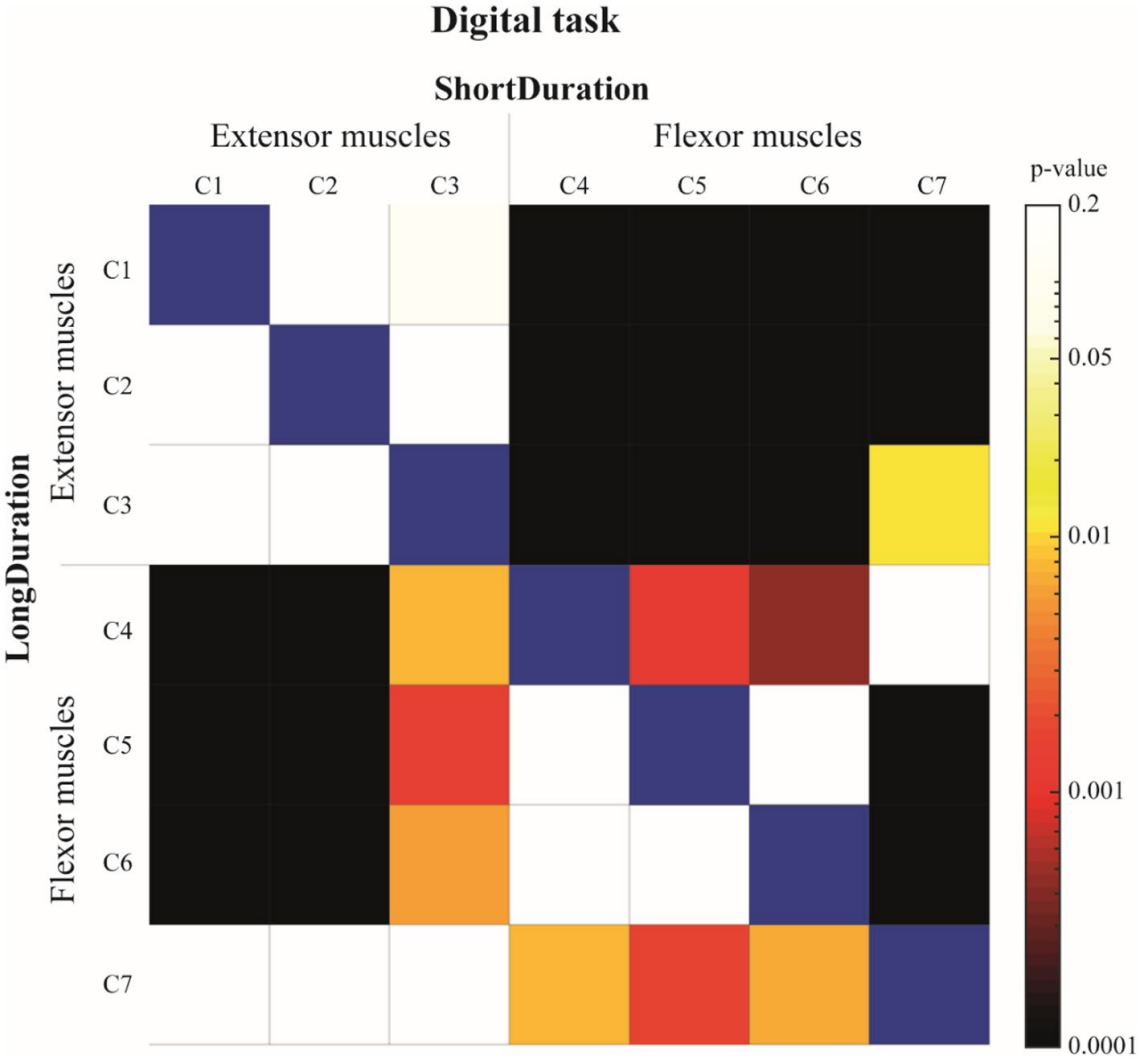
Colormap representation of p values of the Tukey post-hoc analysis for the columns’ comparison of the *Digital* task. Results of the *ShortDuration* group are represented above the blue diagonal while results of the *LongDuration* group are represented below the blue diagonal (α = 0.05). For example, the variation of EMG median frequency (I_5_–I_0_) of *c1* for the *ShortDuration* group (first row, first column) is not significantly different from *c2* (first row, second column), but is significantly different from *c4* (first row, fourth column). Similarly, the variation of EMG median frequency (I_5_–I_0_) of *c1* for the *LongDuration* group (first column, first row) is not significantly different from *c2* (first column, second row), but is significantly different from *c4* (first column, fourth row).

There was a significant main effect of Column on the variation (I_5_–I_0_) of EMG median frequency at the task termination. Post-hoc analysis revealed a significantly greater decrease of EMG median frequency in most of the extensor muscles compared to flexor muscles for the *ShortDuration* and *LongDuration* groups (Fig. 4).

#### Chord task

**Figure 5:**
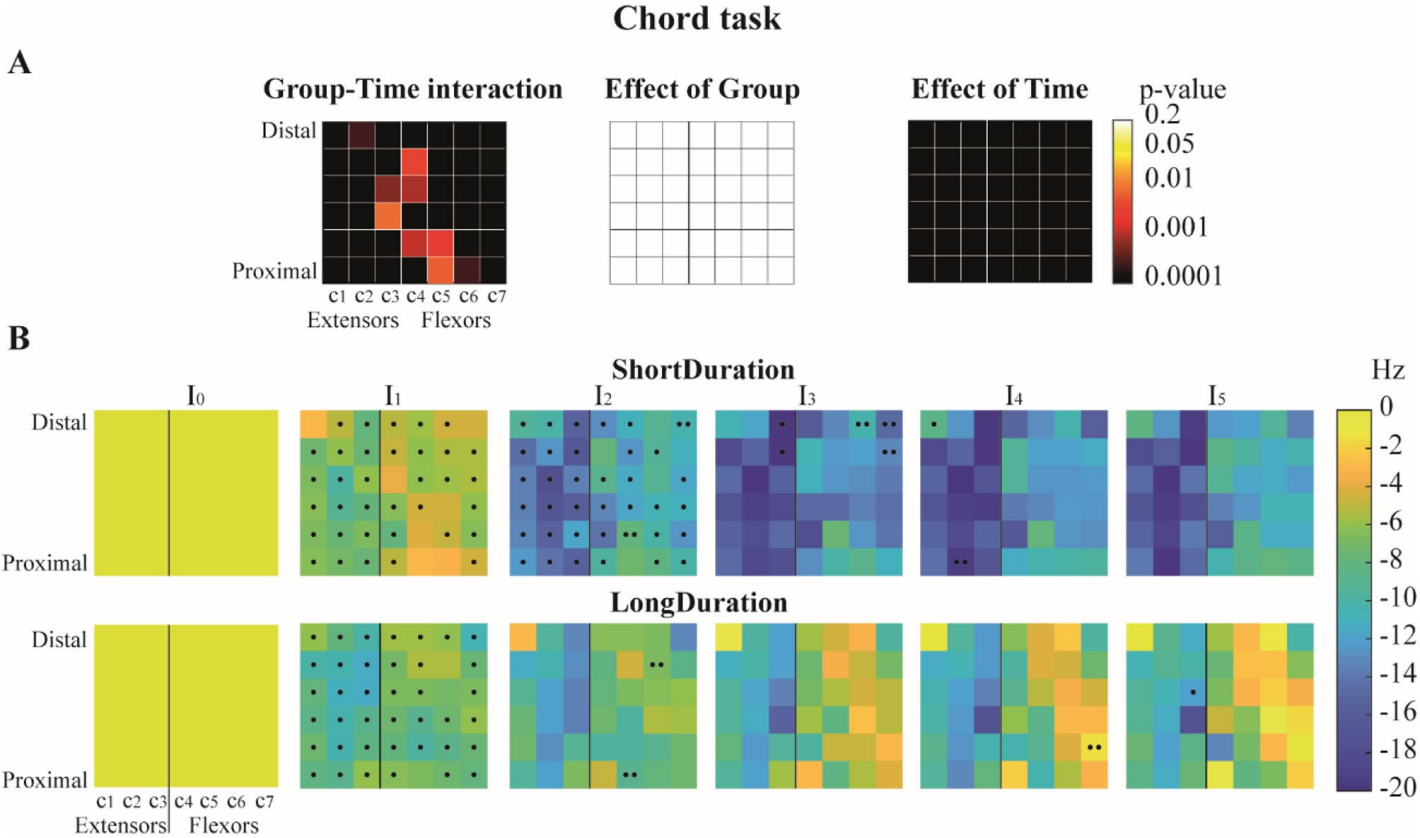
(**A**) colormap representation of ANOVA results’ p values for Group–Time interaction, main effects of Group, and Time for the *Chord* task. Columns of electrodes are labelled *c1* to *c7* as illustrated on the Fig. 8B. (**B**) Average change from baseline (I_0_) representation of the participants’ EMG median frequency of the forearm muscles throughout the duration of the task (I_0:5_), for the *ShortDuration* (superior panel) and *LongDuration* (inferior panel) groups. Note that ANOVA were performed on EMG median frequency values and that variation from baseline was for representation only. Dots indicate significant differences revealed by post-hoc analyses. One dot indicates a significant difference between time intervals I_i_ and I_i-1_ for a given pair of electrode, while two dots indicates a significant difference between time intervals I_i_ and I_i−2_. For example, at time interval I_4_ for the *ShortDuration* group, the dot on the left column (*c1*) of the top row indicates a significant difference between the EMG median frequency at time intervals I_3_ and I_4_, while the two dots on the second column (*c2*) of the last row indicates a significant difference between the EMG median frequency at time intervals I_2_ and I_4_.

There was a significant Group–Time interaction for the EMG median frequency in all the forearm bipolar signals (Fig. 5A). The median frequency decreased for both groups over time, with a greater decrease in the *ShortDuration* group (ES = 1.07) than the *LongDuration* group (ES = 0.18). Post-hoc analyses revealed that for *ShortDuration* group, median frequency significantly decreased from I_0_ to I_1_ and from I_1_ to I_2_ for most of the bipolar signals. For the *LongDuration* group, median frequency significantly decreased between I_0_ and I_1_ for most of the bipolar signals, and remained mostly unchanged during the rest of the task (Fig. 5B; Fig. 3 in Appendix-A4).

**Figure 6:**
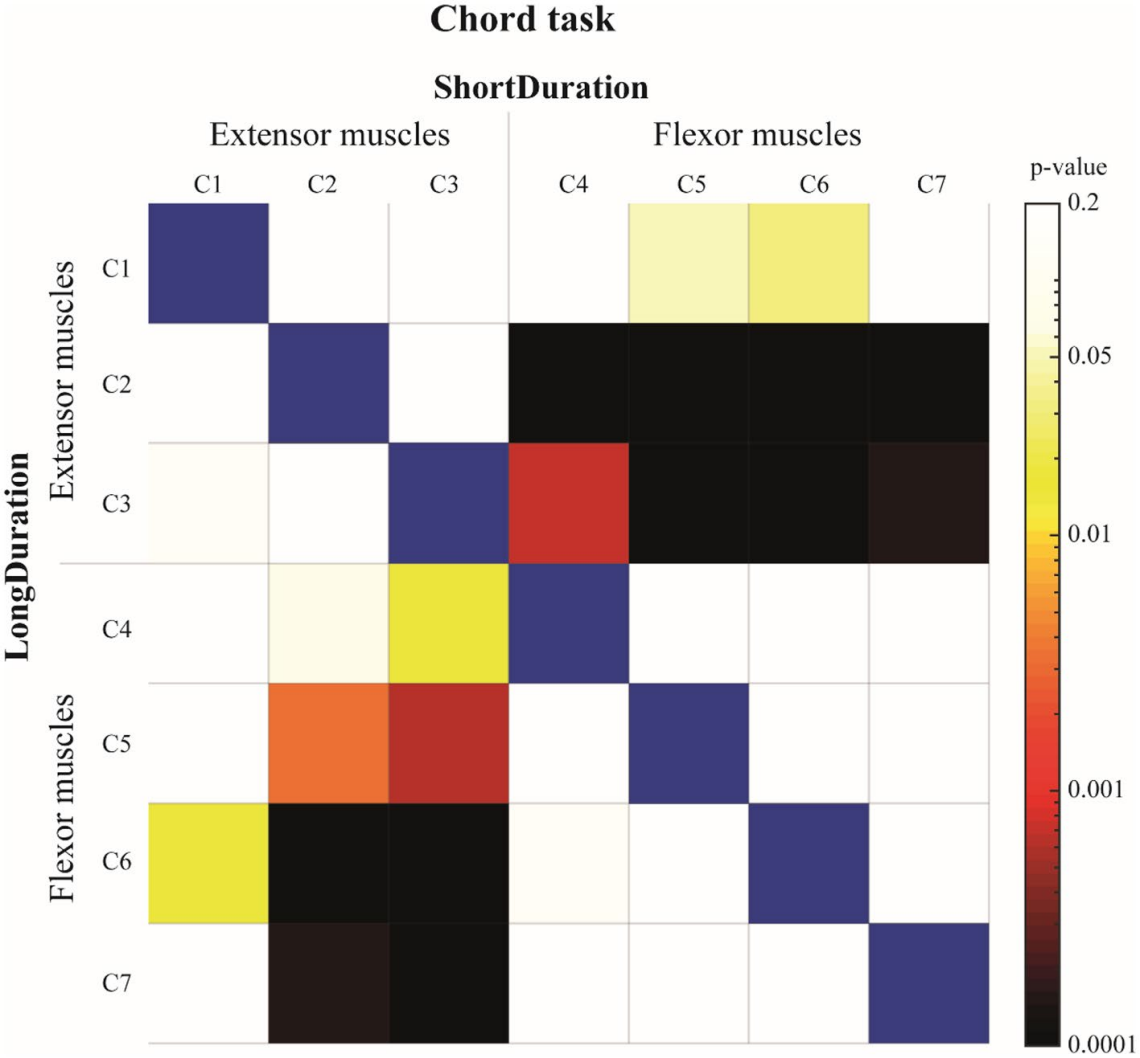
Colormap representation of p values of the Tukey post-hoc analysis for the columns’ comparison of the *Chord* task. Results of the *ShortDuration* group are represented above the blue diagonal while results of the *LongDuration* group are represented below the blue diagonal (α = 0.05). For example, the variation of EMG median frequency (I_5_–I_0_) of *c1* for the *ShortDuration* group (first row, first column) is not significantly different from *c2* (first row, second column), but is significantly different from *c5* (first row, fifth column). Similarly, the variation of EMG median frequency (I_5_–I_0_) of *c1* for the *LongDuration* group (first column, first row) is not significantly different from *c2* (first column, second row), but is significantly different from *c6* (first column, sixth row).

There was a significant effect of Column on the variation (I_5_–I_0_) of EMG median frequency at the task termination. Post-hoc analysis revealed a significantly greater decrease of EMG median frequency in most of the extensor muscles recorded by *c2* and *c3* columns of electrodes compared to flexor muscles, respectively for the *ShortDuration* and *LongDuration* groups (Fig. 6).

### Piano performance parameters

#### Digital task

**Figure 7:**
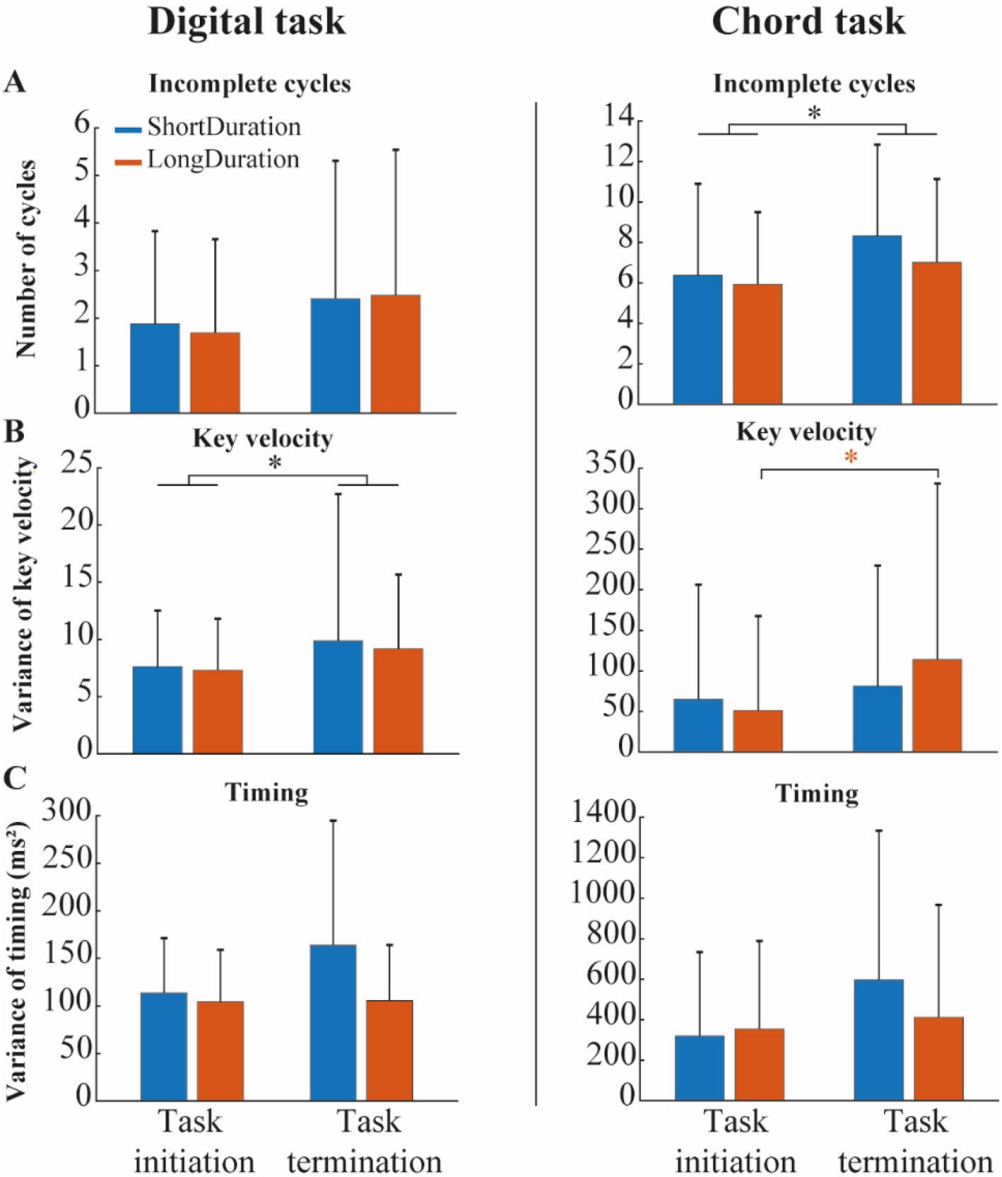
Mean ± standard deviation of (**A**) incomplete cycles, (**B**) key velocity variance, and (**C**) timing variance, for the *Digital* task (left panel) and the *Chord* task (right panel). The black * indicate a significant main effect of Time between the initiation and the termination of each repetitive piano task (α = 0.05). The red * indicates a significant difference only for the *LongDuration* group after post-hoc analysis (α = 0.05).

There was no Group–Time interaction (F(1) = 0.08, p = 0.78) and no main effects of Group (F(1) = 0.01, p = 0.93) and Time (F(1) = 2.07, p = 0.16) on the number of *incomplete cycles* (Fig. 7A, left panel). There was no Group–Time interaction (F(1) = 0.23, p = 0.63) and no main effect of Group (F(1) = 1.23, p = 0.27) on *key velocity* variance, but there was a significant main effect of Time (F(1) = 24.74, p < 0.001, ES = 0.19). *Key velocity* variance was higher during the task termination compared to the task initiation (Fig. 7B, left panel). There was no Group–Time interaction (F(1) = 2.42, p = 0.13) and no main effects of Group (F(1) = 2.76, p = 0.10) and Time (F(1) = 2.61, p = 0.11) on the *timing* variance (Fig. 7C, left panel).

#### Chord task

Participants of both groups performed significantly more *incomplete cycles* during the task termination compared to the task initiation (F(1) = 13.43, p < 0.001, ES = 0.35) (Fig. 7A, right panel). There was no Group-Time interaction (F(1) = 1.11, p = 0.30) and no main effect of Group (F(1) = 0.58, p = 0.45) on the number of *incomplete cycles*. There was a significant Group–Time interaction (F(1) = 4.29, p = 0.04) on the *key velocity* variance. The latter increased for both groups over time (ES = 0.22), with a greater increase for the *LongDuration* (ES = 0.31) than the *ShortDuration* group (ES = 0.11) (Fig. 7B, right panel). Tukey post-hoc analysis revealed that *LongDuration* group had a higher *key velocity* variance during the task termination compared to the task initiation (Fig. 7B, right panel). There was no Group–Time interaction (F(1) = 1.33, p = 0.25) and no main effects of Group (F(1) = 0.35, p = 0.56) and Time (F(1) = 3.12, p = 0.08) on the *timing* variance (Fig. 7C, right panel).

## Discussion

The objective of the present study was to investigate the evolution of forearm muscle fatigue during two repetitive piano sequences and to assess how muscle fatigue alters piano performance. During the *Digital* and *Chord* excerpts, two groups were discriminated based on the time-to-task termination We found that signs of MMF were greater in the *ShortDuration* than the *LongDuration* group and that in both groups, signs of MMF were more important in finger and wrist extensors in both *Digital* and *Chord* tasks. The note-event errors illustrated by *incomplete cycles* tended to increase at the end of both tasks with a significant effect in the *Chord* task. The *key velocity* variability, related to the intensity of the sound, was higher at the end of both tasks.

The time-to-task termination varied between 1 and 12 min in *Digital* task and between 2 and 12 min in *Chord* task. Such variations in time-to-task termination were previously observed^49,50^. Interestingly, the large sample size in this study allowed clustering participants into two groups to assess differences in the evolution of signs of MMF according to the time-to-task termination. Although, there were no significant Group effect in terms of years of experience, practice hours per day, gripping force, age, as well as piano performance parameters at the beginning of each excerpt (Table 1 in Appendix-A3), descriptors of fatigue evolution, i.e., EMG median frequency and mRPE, showed Group–Time interactions. The EMG median frequency decreased quickly for the *ShortDuration* group during the first half of the task (I_2_/I_3_), followed by a slower decrease until the end, especially for the *Digital* task. This pattern was amplified for the *LongDuration* group that showed a decrease of EMG median frequency mainly at the beginning (I_1_–I_2_) followed by constant values of EMG median frequency during both tasks. Although it is well accepted that EMG median frequency linearly decreases during fatiguing contractions until task termination^10,11^, this biphasic behavior of MMF was already shown via the decrease of motor unit discharge rate until 40% of time-to-task termination and then followed by a reversal pattern^51^. While several factors may explain this biphasic mechanism such as low-to-moderate contractions during the fatiguing task or muscle specificities^51^, the evolution of EMG median frequency in our study also suggests that signs of MMF may depend on participant-specific endurance capacities. This results highlights the benefit of sub-grouping participants based on their time-to-task termination to better understand neuromuscular processes underlying fatigue. Interestingly, the evolution of EMG median frequency concorded with the evolution of participants’ effort perception. Indeed, mRPE of *ShortDuration* group increased quickly and constantly during the tasks. On the other hand, mRPE of *LongDuration* group increased during the first 20% and 30% of the tasks (I_2_ and I_3_), respectively for the *Digital* and *Chord* tasks, before remaining constant until the end. This observation strengthens the adequacy between the perceived effort and EMG median frequency evolution showed in previous studies^11–13,19^. Finally, we could hypothesize that for the same amount of time played, pianists with short time-to-task termination would be at a higher risk of developing PRMDs by an increased muscle fatigue accumulation. Therefore, a higher prevalence of PRMDs could be expected in the *ShortDuration* group. A follow-up in a longitudinal study of the sample of pianists who participated to this study could answer this hypothesis.

Muscle fatigue was previously proposed as a risk factor of PRMDs^1,2^, with a higher prevalence reported at the forearm and wrist^7–9^. The presented results of the *Digital* and *Chord* tasks showed that extensor muscles were more prone to show signs of MMF than flexor muscles. This observation supports the idea of a higher prevalence of injury in the extensor muscles. The most common injury among pianists is lateral epicondylitis^7,8^ which is associated with overuse of the wrist extensor muscles. This result is in agreement with large amount of stress undergone by the extensor muscles^7,52^, and a greater strain on the lateral side of the elbow joint during the repetition of piano sequence^52–54^. In addition, previous results suggested that muscle force of wrist flexors was higher than wrist extensors^55–57^. Flexor muscles are used to depress keys in the same direction of gravity force, while extensor muscles are used both as wrist stabilizer and to release keys against gravity. Each change in movement direction requires co-activation of agonist–antagonist muscle pairs, and because wrist flexors have a greater moment arm than the extensor muscles, larger forces are required by the extensor muscles to maintain the wrist posture^53^, which can lead to increase fatigue in this muscle group^53,58^. In line with this idea, our results suggest that pianists’ wrist and finger extensor muscles located at the forearm, which have an antagonist role in relation to key depression and an agonist role in relation to key release, are more prone to develop muscle fatigue in the context of fast and loud *Digital* sequences. The metacarpophalangeal joint is the most important finger joint producing the vertical motion of the fingertip during *Digital* piano task^59^. Consequently, the latters suggested that finger kinematic patterns to press the keys indicated a predominant use of intrinsic hand muscles. Higher contribution on finger flexors muscles located at the hand rather than at the forearm might in part explain the greater signs of MMF in extensor muscles during the *Digital* task. During *Chord* sequences, multi-joint upper-limb movements used to perform loud chords are similar to those of isolated loud tones, where fingertip downward velocity is produced first by elbow extension and then wrist flexion^60^. During a *Chord* piano task, wrist flexors have an agonist role in the depression of keys, while wrist extensors act as continuous stabilizers of this joint^7^. In our study, wrist flexion probably contributed to both loudness and fast repetition of chords at the middle-high and high registers. However, as for the *Digital* task, the reported evolution of the median frequency during the *Chord* task suggested a higher incidence of muscle fatigue in extensor muscles, which is probably related to their continuous stabilizer role.

Concerning musical performance, fatigue, as evidenced by mRPE and EMG median frequency at task termination, significantly altered piano performance parameters. This result support previous findings showing a detrimental effect of fatigue on performance in wind instrumentalists as well as during sport activities, postural adjustment task, or circular horizontal arm movement^40,41,46,61–63^. In our study, *key velocity* variance increased in both *Digital* and *Chord* tasks. As *key velocity* is directly related to the intensity of the sound produced^37^, our results indicate that muscle fatigue might negatively affect precise control of sound intensity levels. This result is in accordance with the increase of finger force variability in discrete and cyclic force production tasks performed by all four fingers (excluding the thumb) pressing in parallel^64^. This increased variability in fingers’ force production could be a strategy to prevent or slower the development of muscle fatigue as previously suggested^65–68^, and could contribute to the increased variability in *key velocity* observed in our study.

Although mean values related to note-event accuracy tended to be negatively affected by fatigue in both *Digital* and *Chord* tasks, fatigue effect was only significant during the *Chord* task. Regardless of their group classification, pianists performed significantly more incomplete cycles at the termination of the *Chord* task than at the initiation. An undershoot of the final position previously reported in extension movement under muscle fatigue condition^42^ could be related to note-event accuracy. This result reflects that muscle fatigue affects the accuracy of large amplitude movements such as those in the *Chord* task involving big leaps across different registers and the execution of fast repetitions of the same notes. The non-significant effect of fatigue in the *Digital* task may be due to smaller hand range of motion and/or slower rhythm in the excerpt investigated (0.13 s vs. 0.094 s between notes, respectively in the *Digital* and *Chord* tasks). Consequently, the effects of fatigue on note-event accuracy may be influenced by the musical style of the selected piece, and further investigation of this aspect should be explored.

Although mean values of *timing* variability tended to be higher at the end of the *Digital* and *Chord* tasks, absence of a significant effect of fatigue suggests that pianists’ control of the inter-onset timing between successive notes is less affected by fatigue than sound intensity and accuracy. Taken together, our results highlight that piano performance parameters were negatively affected by fatigue. Future studies should focus on investigating additional musical parameters that could be affected by fatigue, such as articulation and rhythmic errors.

Our study has some limitations. While the *Digital* and *Chords* tasks were based on excerpts from the piano repertoire (*Chord* task) and an exercise designed to target independent finger activity (*Digital* task) commonly used in pianists’ training^69,70^, their continuous repetition until exhaustion is not representative of typical piano practice. However, this choice allowed to standardise piano performance parameters among participants as in previous studies on the biomechanics of piano performance^24,37^. The arrangement of EMG electrodes is a second limit as the forearm is composed of 20 muscles difficult to record individually. However, we believe that the standard procedure used to place electrodes diminished the precision recording bias. Moreover, it allowed localising forearm area where signs of MMF is more prone to develop (i.e., posterior area). Future studies could then increase the spatial sampling in this particular area to increase precision. Another limitation is that only finger flexion MVC was measured via the handgrip task while finger extension MVC, which may differ between the *ShortDuration* and *LongDuration* groups, was not assessed. However, previous studies showed that extensor muscles are highly active during a handgrip test^71^, and that a correlation exists between the isometric and isokinetic torques recorded during wrist flexion and wrist extension^72^ that may suggest that both groups had similar wrist flexion and extension MVC. Finally, it must be mentioned that the 60–120–180 Hz ± 1 Hz stop-band second order zero-lag Butterworth filters used to remove 60 Hz electrical contamination and its harmonics also removed EMG components. However, the removal of this narrow components, i.e. 6 Hz out of the 10–400 Hz frequency band of interest of EMG signals, marginally affects the EMG power spectrum as previously shown in Mello et al.^73^. Consequently, this pre-processing step should not have a significant impact on the changes in EMG median frequency as the power spectrum shift towards lower frequencies in conditions causing muscular fatigue would affect all EMG frequency components.

In conclusion, this study showed that continuous repetition of different piano excerpts leads to muscle fatigue. As muscle fatigue caused by repetitive motion of piano could be a precursor of PRMDs, finger and wrist extensors muscles seem more subjected to risk of injuries than flexors muscles, as suggested in epidemiological studies. Finally, muscle fatigue developed through the repetition of movement patterns affected note-event and key velocity musical performance parameters among expert pianists.

## Methods

### Participants

Fifty expert pianists volunteered to participate in this study. All participants had at least a university (or equivalent) degree or were enrolled in undergraduate or graduate studies in piano performance. Forty three of them were right-handed. Participants were not included if they reported any PRMDs during the year preceding the experimentation. Their characteristics are described in Table 1 (“Results” section). After receiving instructions on the experimental protocol, participants read and signed a written informed consent. The protocol was approved by the Université de Montréal’s Ethics Committee (CPER-18-086-D), and all the study was performed in accordance with the STROBE guidelines.

### Instrumentation

**Figure 8:**
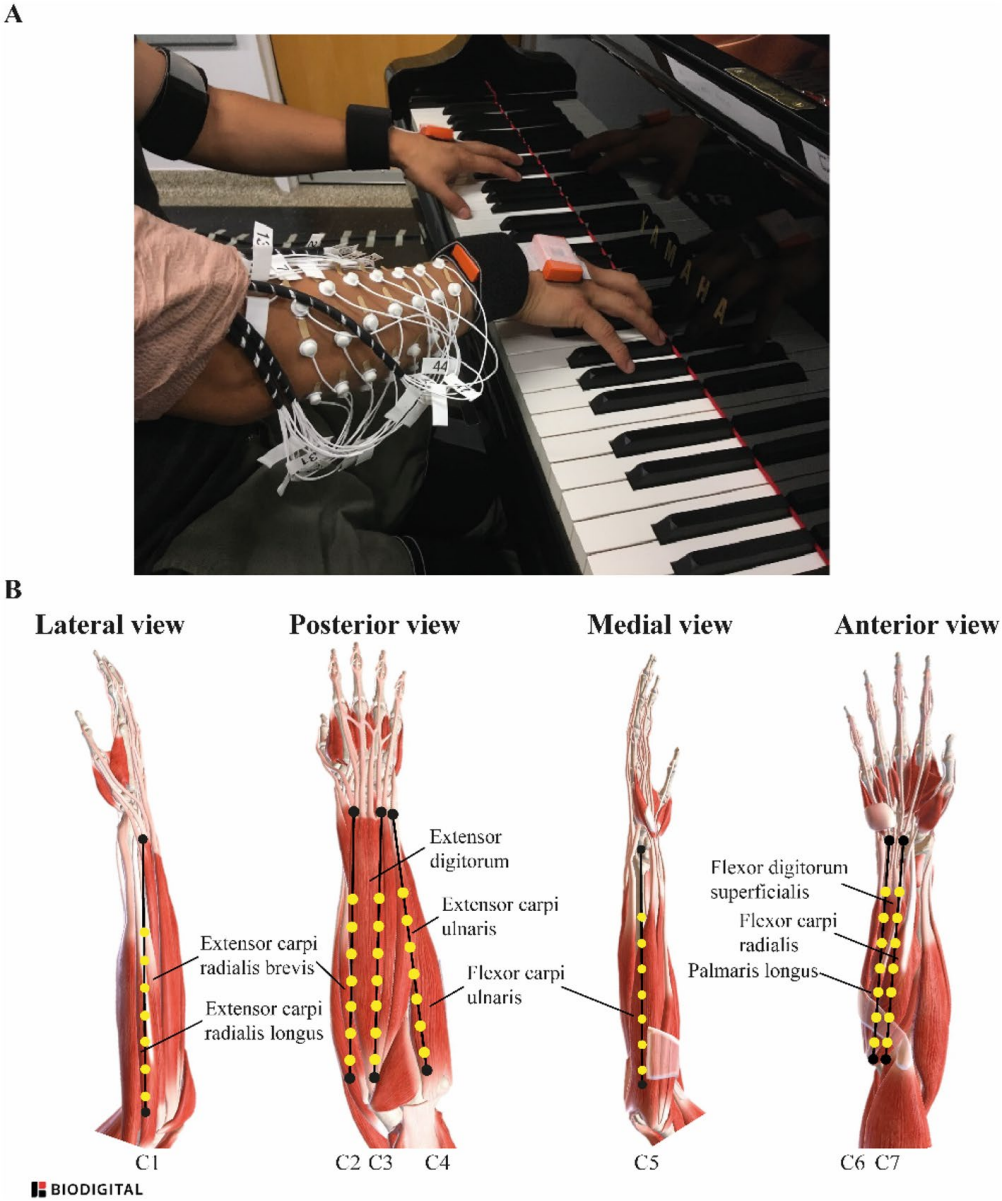
(**A**) close-up of the electrodes positioned on the forearm, and (**B**) schematic view of the 7 × 7 array of electrodes positioned on the forearm and the underlying muscles. Forearm views were generated through BioDigital and electrode positioning was drawn by the authors.

Participants were equipped with 49 monopolar EMG electrodes of 1.5 mm diameter (TMSi, EJ Oldenzaal, The Netherlands) positioned on the right forearm muscles according to a 7 × 7 array as shown in Fig. 8. Previous studies showed that the hotspot of EMG activation of the forearm depends on the task performed. During wrist extension, radial and ulnar deviation, as well as individual four finger extensions at the metacarpophalangeal joint^74,75^, EMG activation hotspots were distributed over a large surface of the forearm. All these contractions are made by pianists while performing. Therefore, the 49 monopolar EMG electrodes covered the entire forearm. Before electrode placement, forearm skin was scrubbed with 70% isopropyl alcohol pads. A conductive gel was used to improve skin-electrodes conductivity. Electrodes were attached to the skin with circle double-sided tape. Additionally, a medical elastic net bandage (not shown in Fig. 8) was used to avoid electrode detachment. Electrodes were positioned 2 cm apart along columns, which were arranged on the forearm as follows: *c1* from the lateral edge of the brachio-radial muscle to the midpoint between the base of index and middle fingers; *c2* from the medial-inferior edge of the radial head to the midpoint between the base of middle and ring fingers; *c3* from the lateral-inferior edge of the radial head to the lateral-posterior edge of the ulnar head; *c4* from the medial edge of the ulnar crest to the medial-anterior edge of the ulnar crest; *c5* from the medial-posterior epicondyle to the posterior pisiform; *c6* from the medial-anterior epicondyle to the anterior pisiform; and *c7* from the medial edge of the bicipital tendon to the anterior aspect of the base of the second metacarpal. The electrode positioning procedure was performed by a trained physiotherapist to ensure reliability and physiological meaning of the data. EMG signals were acquired using a 72-channel Refa amplifier (TMSi, EJ Oldenzaal, The Netherlands) at a sampling rate of 2048 Hz.

An instrumented grand piano (Disklavier DC7X Enspire Pro, Yamaha Canada Music Ltd, Toronto, Canada) was used to record key depression timing and hammer velocity of each key. A digital sound level meter (The 407730 SLM, Extech Instruments, Nashua, USA) was placed at a 1.5 m distance of the back-left side of the piano bench to continuously monitor sound intensity level.

### Experimental procedures

Participant’s maximal handgrip force was measured using a handgrip dynamometer (Takei Scientific Instruments, Tokyo, Japan). They performed two 5-s maximal voluntary contractions with their right hand interspaced with a 1-min rest period. Verbal encouragement were provided during contractions.

After a 5-min warm-up piano session, a sound test was performed to familiarize participants with the required sound intensity level. They were then asked to play on a loop without interruption two excerpts named *Digital* and *Chord* tasks described thereafter. Both excerpts were played fast and loud to increase muscle activation levels^7,24,76,77^ to accelerate the development of muscle fatigue. The music score of the *Digital* and *Chord* excerpts were sent to participants at least 5 days prior to the experiment so that they practice them in order to be able to perform them comfortably. The *Digital* task corresponded to a 16-tone right-hand digital sequence on the first two measures of Exercise no 7 from C.L. Hanon’s ‘The Virtuoso Pianist’ spanning a major sixth interval in the middle register of the keyboard (Appendix-A1). Participants were asked to play this excerpt loud (minimum threshold = 78 dB) and fast (quarter note = 112 BPM [beats per minute]) and with no accents in the melody. The *Chord* task corresponded to a single-chord right-hand sequence on the measure 119 of Franz Liszt’s Ballade no 2 in B minor S.171. The chord was composed of three notes repeated five times: once in the middle register, fast repetition in the middle-high register, and fast repetition in the high register of the keyboard (Appendix-A1). Participants were asked to play this excerpts loud (minimum threshold = 84 dB [middle register] and 94 dB [high register]) and fast (quarter note = 120 BMP) while conserving dynamics and musical intention.

The order of the excerpts was randomly assigned and interspaced with a 15-min rest period. Participants wear an earphone in their left ear that indicated the tempo. A screen was placed in front of the piano to inform participants if sound intensity levels were lower than the minimum loudness threshold. Finally, the rate of perceived exertion was monitored every 30-s via the modified CR-10 Borg scale (mRPE)^48^ displayed on the screen. Participants were stopped if they reached twice in a row a score of 7 or after 12 min of continuous playing^78^.

### Data processing

Data processing and statistical analyses were performed with Matlab R2019a (The MathWorks Inc., Natick, MA, USA).

#### Perceived fatigue

The mRPE scores of participants were time normalized into 100 values, before selecting data of six intervals, namely I_0_: 0–10%, I_1_: 10–20%, I_2_: 30–40%, I_3_: 50–60%, I_4_: 70–80%, and I_5_: 90–100% of the total duration time of each participant.

#### Electromyography analysis

Monopolar EMG signals were converted into bipolar EMG signals by subtracting the two closest monopolar signals along columns. EMG data were then filtered using a 10–400 Hz band-pass filter and a 60-120-180 Hz ± 1 Hz stop-band filter to remove 60 Hz electrical contamination and its harmonics. All filters were second order zero-lag Butterworth filters. Data were then zero-aligned by subtracting the mean value, and finally down sampled to 1024 Hz to reduce the time of computation in the subsequent time–frequency analyses. A time–frequency analysis was performed by applying a continuous Morlet wavelet transform (wave number: 7, frequency range: 1–400 Hz in 1 Hz steps) to the pre-processed EMG signals (WavCrossSpec Matlab package^79^). The instantaneous EMG median frequency was then computed on a time-history basis. The mean of the instantaneous median frequency was calculated for each cycle determined using key depression timing. A cycle refers to each repetition of *Digital* and *Chord* excerpts. Data were time normalized, and the mean of cycles’ median frequency was calculated during the six intervals described previously. We found 16 EMG bipolar channels out of 2303 (47 muscles × 49 participants) with persistent artifacts in the *Digital* task and 24 EMG bipolar channels out of 2303 with persistent artifacts in the *Chord* task. For each interval of abnormal data, median frequency was set to the median value of the group^80^.

#### Piano performance analysis

Key depression timing and velocity data were used to compute piano performance parameters, which were related to note events, key velocity (a performance feature highly related to sound intensity), and timing (time intervals between the onset of successive notes)^35–38^. Performance parameters described thereafter were computed for the first and the last 30-s of both *Digital* and *Chord* tasks for each participant. The analysis started after the first 10 cycles of each task which were considered as a warm-up period.

*Incomplete cycles* were defined in relation to the notes/chords events. Due to their different musical characteristics, *Digital* and *Chord* tasks were analyzed using a distinct rationale. For the *Digital* task, a cycle was considered as incomplete when participants missed a note or played a wrong note. For the *Chord* task, a cycle was considered as incomplete when participants (1) unsuccessfully played the three notes of the chord in all three registers, independently of the successful repetition of the chord in the middle-high and high registers, and (2) when they unsuccessfully performed the repetitions of the highest note of the chord in the middle-high and high registers as the highest note is considered as the most important note of the chord^81,82^.

*Key velocity* variability was assessed for both *Digital* and *Chord* tasks using the key velocity data of the completed cycles. For the *Digital* task, the *key velocity* variance was calculated for each of the 16 notes of the excerpt. For the *Chord* task, the *key velocity* variance was computed from the velocity of the highest note of each of the 5 chords^81,82^.

*Timing* variability was assessed for both *Digital* and *Chord* tasks using the key depression timing data of the completed cycles. For the *Digital* task, the *timing* variance between the 16 notes of the excerpt was calculated. For the *Chord* task, the *timing* variance of the highest note of the chord played twice in the middle-high and high registers was computed.

### Statistical analysis

Since the time-to-task termination between participants ranged from 1 to 12 min in *Digital* task, and from 2 to 12 min in *Chord* task, we expected different effect of time on the evolution of fatigue and piano performance. Consequently, based on the participants’ time-to-task termination, a k-means clustering and a silhouette validity index were used to identify the optimal number of sub-groups for each task. As described in the Results section, the cluster analysis discriminated two groups named *ShortDuration* and *LongDuration* groups. Consequently, in addition to the effect of time, a Group effect and a Group-Time interaction were considered in subsequent statistical analyses. Group comparison was performed for demographic data using t test or F test when appropriate, and a false discovery rate correction was applied to p values.

In terms of fatigue evolution (mRPE and EMG median frequency), a two-way ANOVA on Group (*ShortDuration* vs *LongDuration*) × Time (I_0_, I_1_, I_2_, I_3_, I_4_, I_5_) with repeated measures on the last factor was performed for mRPE and EMG median frequency of each bipolar EMG signal followed by a Tukey post-hoc analysis when appropriate. In the post-hoc analysis, we focused on the differences between I_i_ and I_i-1_ and between I_i_ and I_i-2_ only when there was no difference between I_i_ and I_i-1_. Finally, to determine the location of the forearm with greater signs of MMF at the end of each task, a one-way ANOVA on Column (*c1*, *c2*, *c3*, *c4*, *c5*, *c6*, *c7*) was performed on the difference of EMG median frequency between I_5_ and I_0_ for both groups and both tasks separately. In case of significant effect, a Tukey post-hoc analysis was performed to assess differences between each pair of column. For this analysis, the variation (I_5_–I_0_) of EMG median frequency was used since the frequency content of the EMG can be affected by factors such as the thickness of the subcutaneous tissues between electrodes and muscles^83^.

In terms of piano performance, a two-way ANOVA on Group (*ShortDuration* vs *LongDuration*) × Time (initiation vs termination) with repeated measures on the factor Time was performed for *incomplete cycles*, *key velocity* variance, and *timing* variance.

For each significant main effect and post-hoc test, Cohen’s *d* effect size (ES) statistic was computed as follows^84,85^:

$$ES=\frac{\stackrel{-}{{X}_{2}}-\stackrel{-}{{X}_{1}}}{pooledSD}$$

With $pooledSD=\sqrt{\frac{{{SD}_{X1}}^{2}*\left({n}_{1}-1\right)+{{SD}_{X2}}^{2}*\left({n}_{2}-1\right)}{{n}_{1}+{n}_{2}-2}}$ for independent samples comparison.

And $pooledSD=\sqrt{\frac{(X1-X2)^{2}}{N-1}}$ for one-sample comparisons.

In this equation, n1 and n2 are the sample size for each condition, N is the total sample size. ES were qualitatively interpreted as huge (ES ≥ 2), very large (1.2 ≥ ES > 2), large (0.8 ≥ ES > 1.2), medium (0.5 ≥ ES > 0.8), small (0.2 ≥ ES > 0.5), very small (0.01 ≥ ES > 0.2) as suggested by Cohen^84,85^.

## Supplementary Information

Supplementary Information 1.

## Abbreviations

HD-EMG: High-density electromyography
mRPE: Modified rate of perceived exertion
MVC: Maximal voluntary contraction
PRMDs: Playing-related muscular disorders
SD: Standard deviation
