# Supplementary material for: Exhausting repetitive piano tasks lead to local forearm manifestation of muscle fatigue and negatively affect musical parameters
Source: Sci Rep. 2021 Apr 14;11:8117. doi: 10.1038/s41598-021-87403-8 (PMC8047012; doi:10.1038/s41598-021-87403-8)
Supplement: Supplementary file 1 — Supplementary Information 1. [file 41598_2021_87403_MOESM1_ESM.docx]

**Title:** Exhausting repetitive piano tasks lead to local forearm manifestation of muscle fatigue and negatively affect musical parameters

**Authors:** Etienne Goubault^a^*, Felipe Verdugo^b,c^, Justine Pelletier^d^, Caroline Traube^e^, Mickaël Begon^a,f^, Fabien Dal Maso^a,g^

^a^Laboratoire de Simulation et Modélisation du Mouvement, École de Kinésiologie et des sciences de l’activité physique, Université de Montréal, 1700 Rue Jacques-Tétreault, Laval, Québec, Canada (affiliation where the research was conducted)

^b^Input Devices and Music Interaction Laboratory, Centre for Interdisciplinary Research in Music Media and Technology, Schulich School of Music, McGill University, Montreal, Quebec, Canada

^c^EXPRESSION Team, Université Bretagne-Sud, Vannes, France

^d^Laboratoire Arts vivants et interdisciplinarité, Département de danse, Université du Québec à Montréal, Montreal, Quebec, Canada

^e^Laboratoire de recherche sur le geste musicien, Faculté de musique, Université de Montréal, Montreal, Quebec, Canada

^f^Sainte-Justine Hospital Research Center, Montreal, Québec, Canada

^g^Centre interdisciplinaire de recherche sur le cerveau et l’apprentissage, Montréal, Québec, Canada

***Corresponding author:** [etienne.goubault.de.brugiere@umontreal.ca](mailto:etienne.goubault.de.brugiere@umontreal.ca)

# Appendices

## A1. Experimental instruction for both Digital and Chord excerpts

***Digital task: the first two measures of the exercise no.7 of ‘The Virtuoso Pianist’ (C.L. Hanon)***


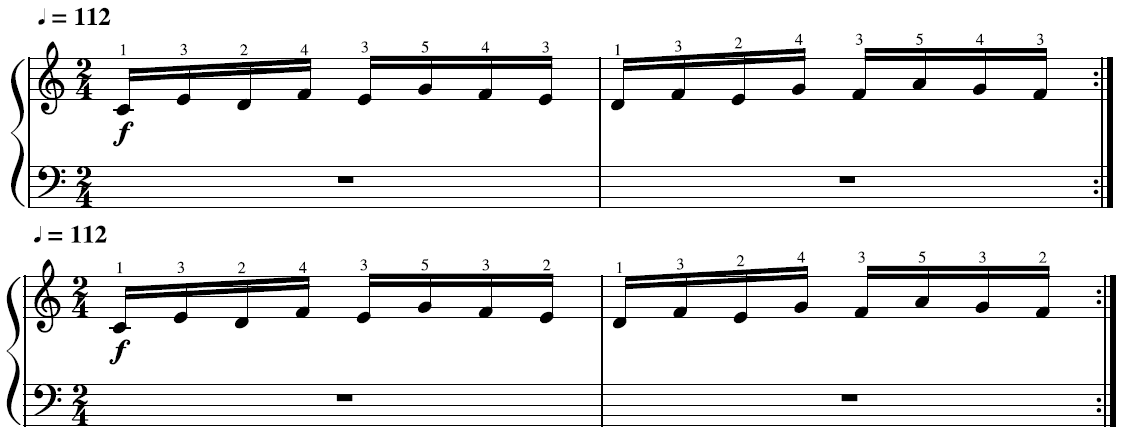


Instructions: *i)* use your right hand only (left hand resting on your left thigh); *ii)* As this excerpt can be performed using different fingering options, participants were asked to choose one fingering between two given options to facilitate standardization of experimental procedures and to allow participant to play comfortably. The chosen fingering options differed at two specific parts of the excerpt and concerned in both cases the annular, the middle, and the index fingers.

***Chord task: the 119th measure of the Ballade no.2 in B minor S.171 (F. Liszt)***

Instructions: *i)* play the bar framed by a rectangle only; *ii)* do not play the left-hand notes crossed by a X; *iii)* hold the sustain pedal from the first beat and release it at the beginning of the fourth beat for each iteration; *iv)* hold the left-hand notes until the third beat (as if they were dotted half notes). A unique and standard fingering option was given to perform this excerpt.

## A2. Example of EMG signals

**A**


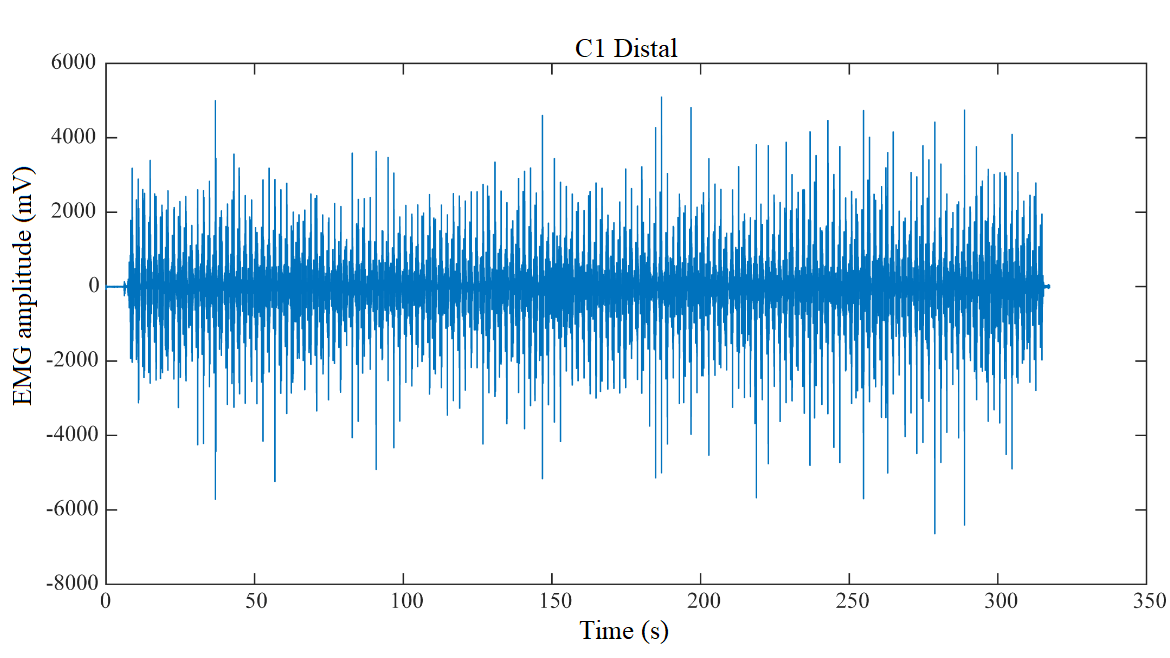


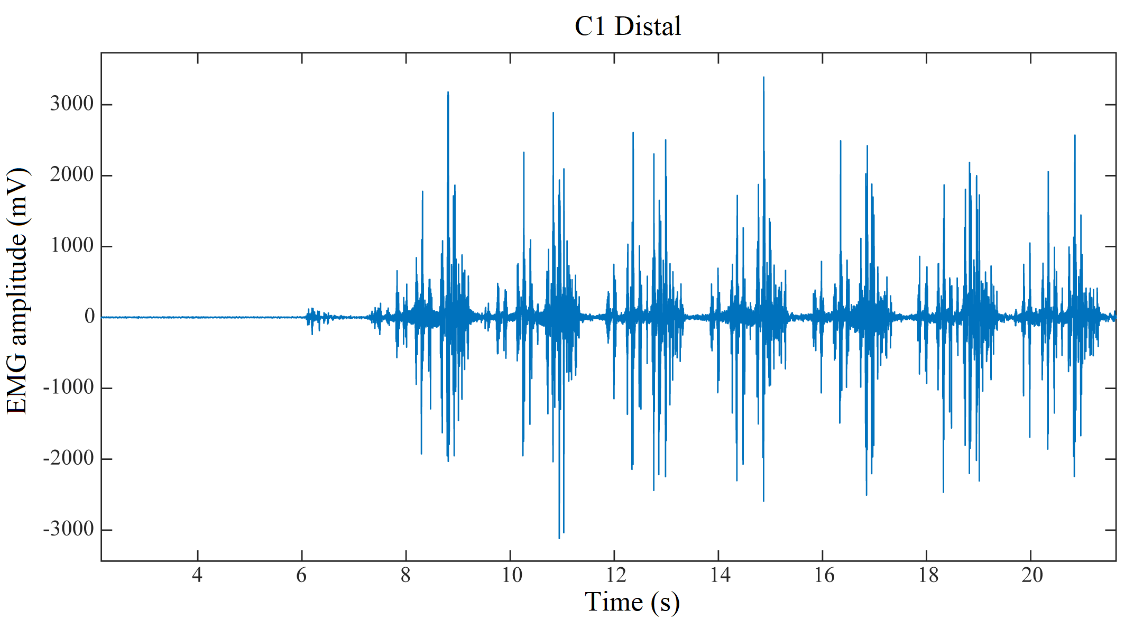


**B**


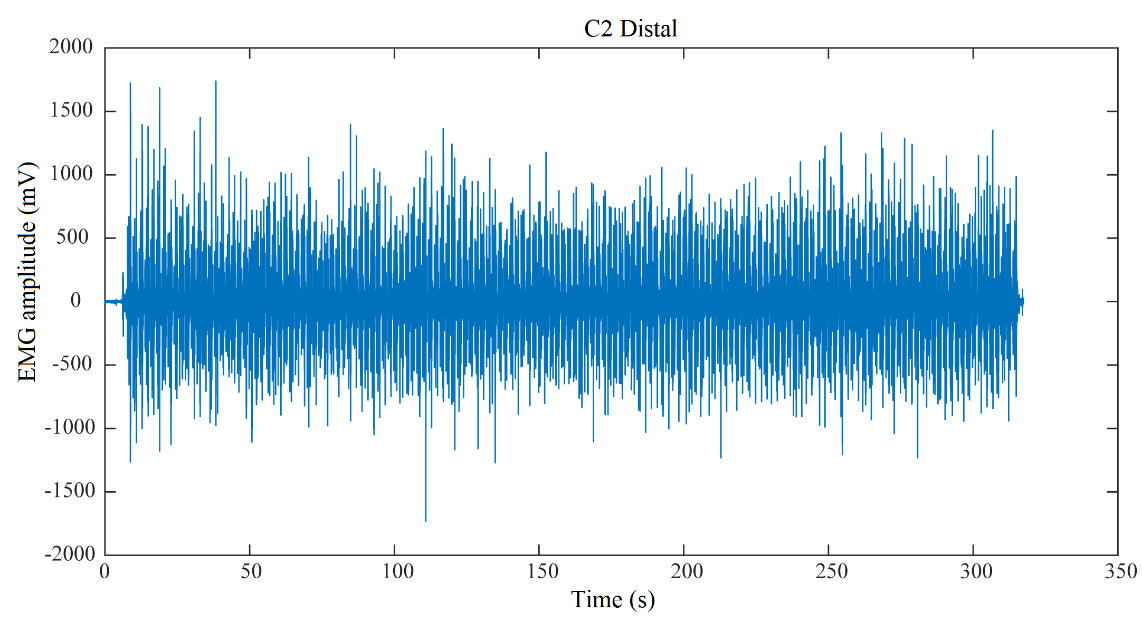


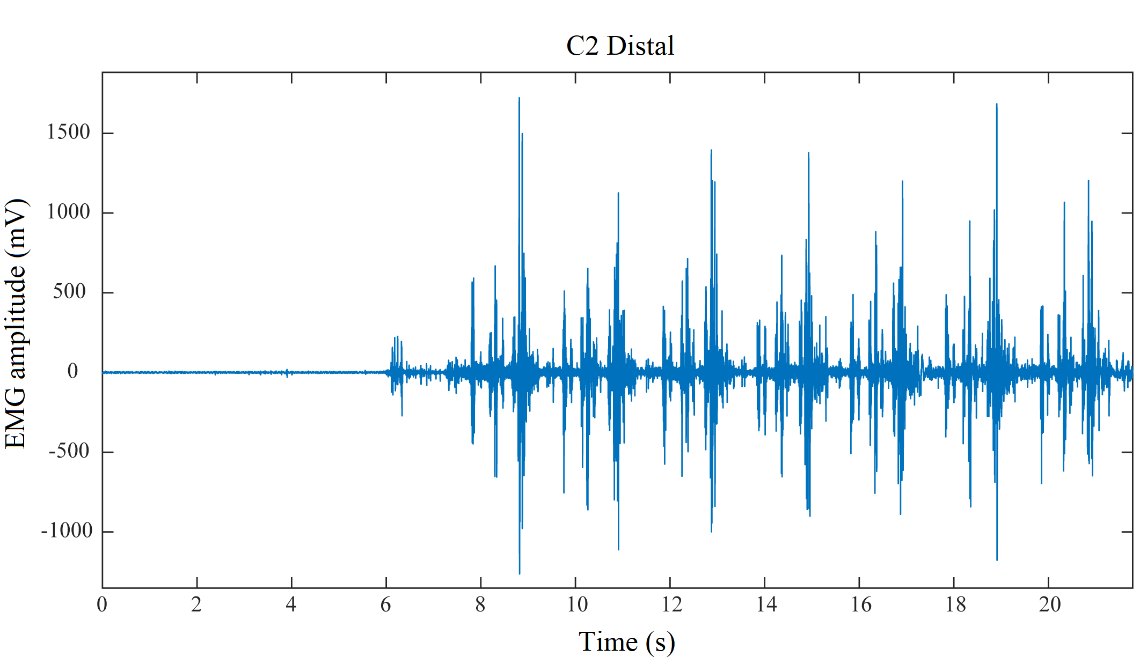


**C**


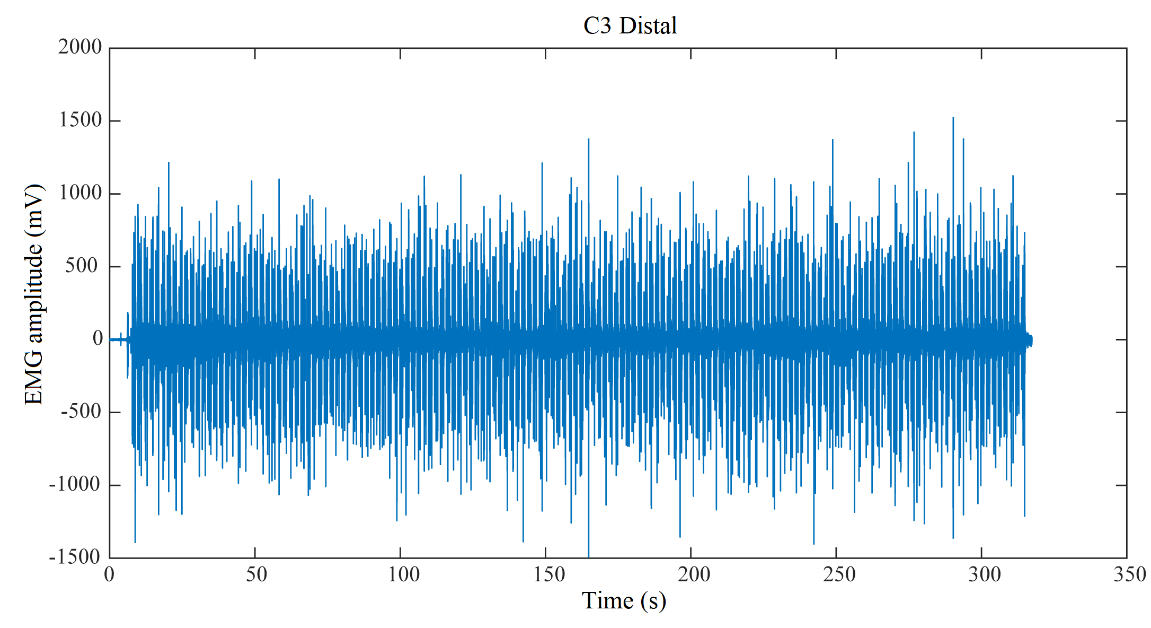

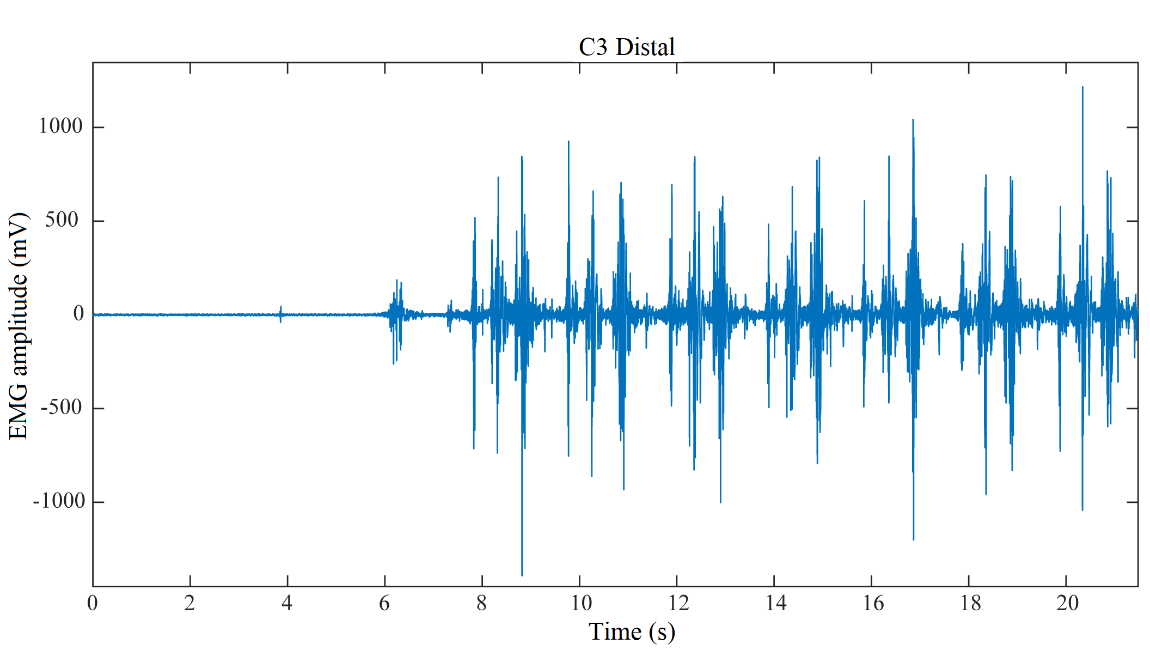


**D**


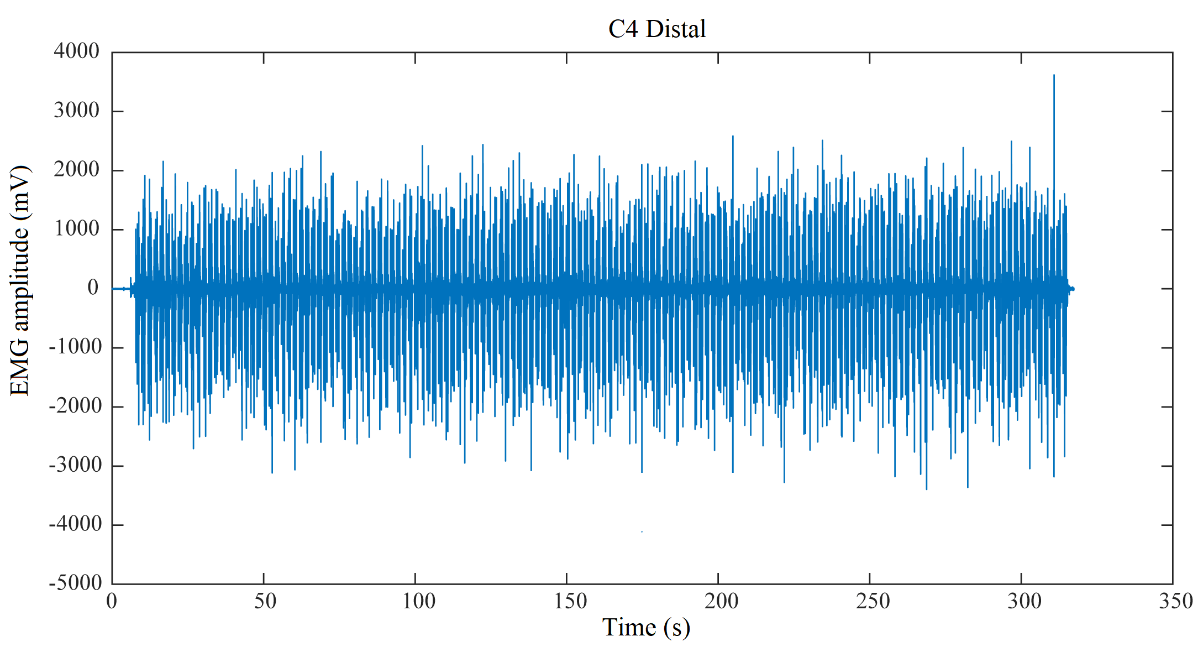

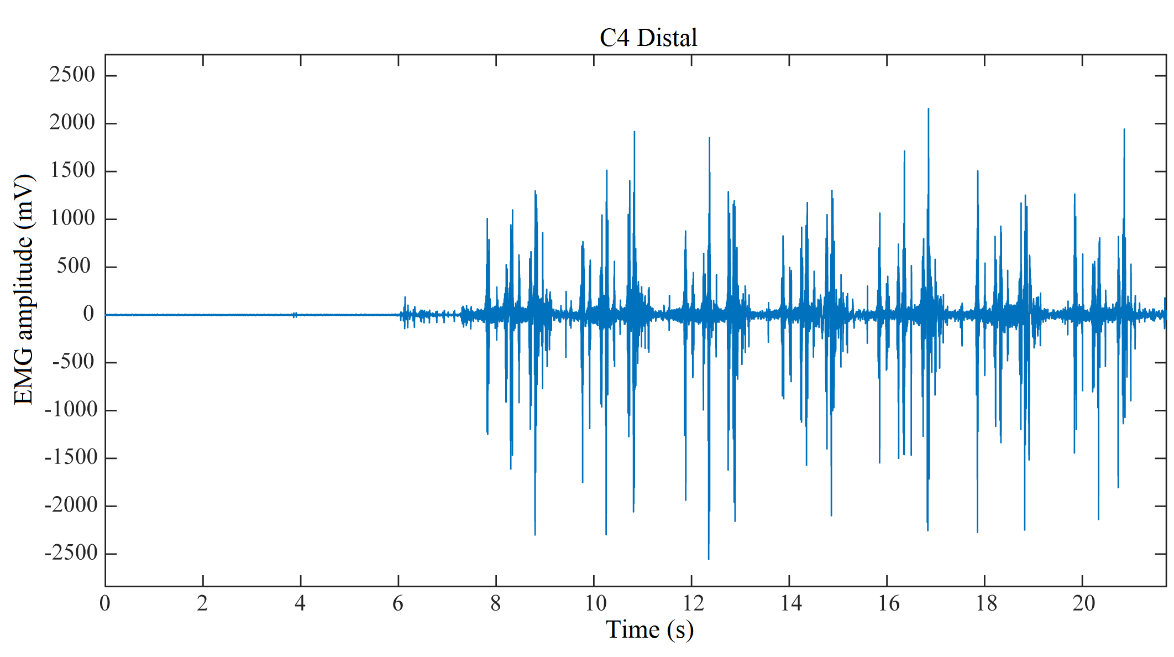


**E**


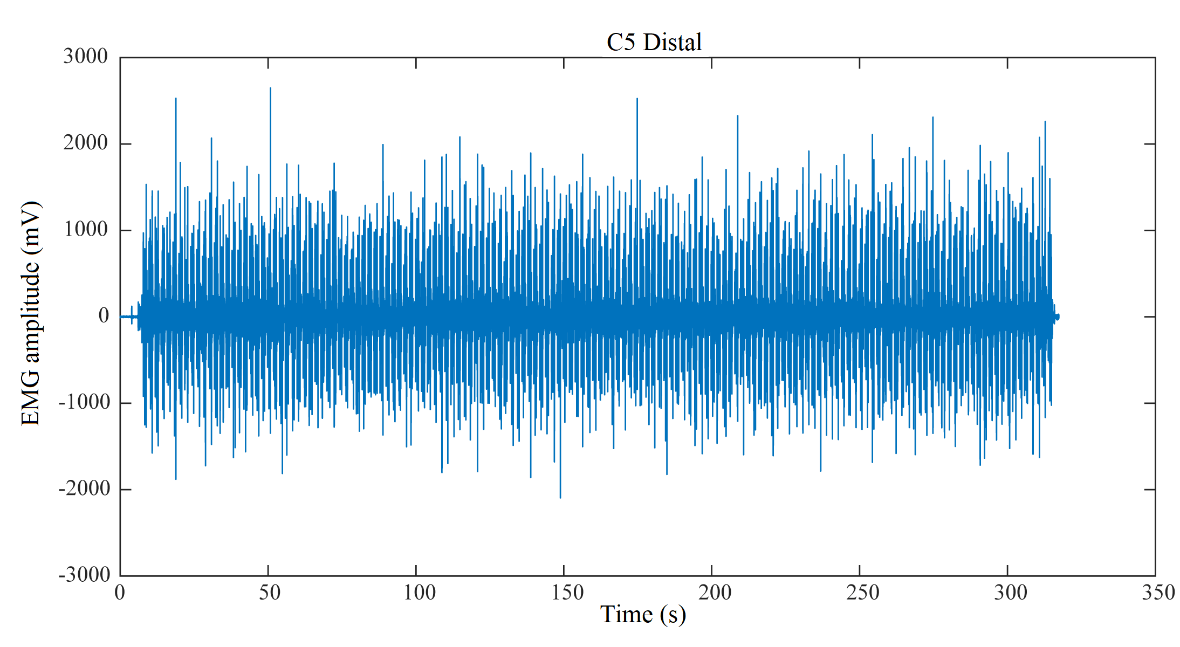

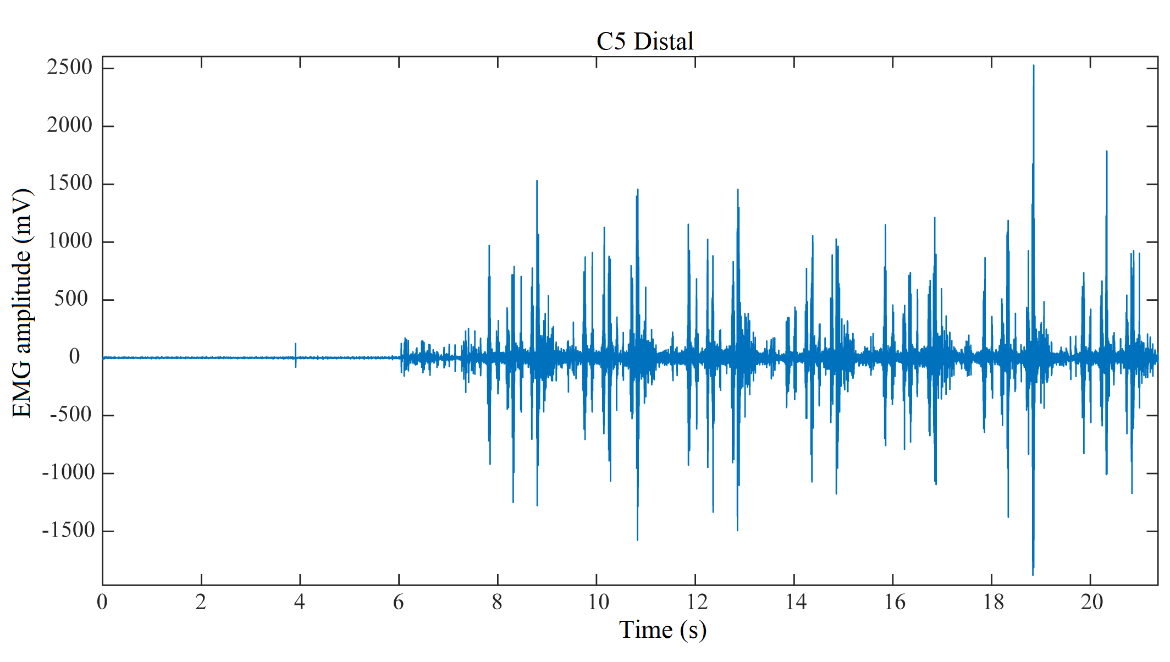


**F**


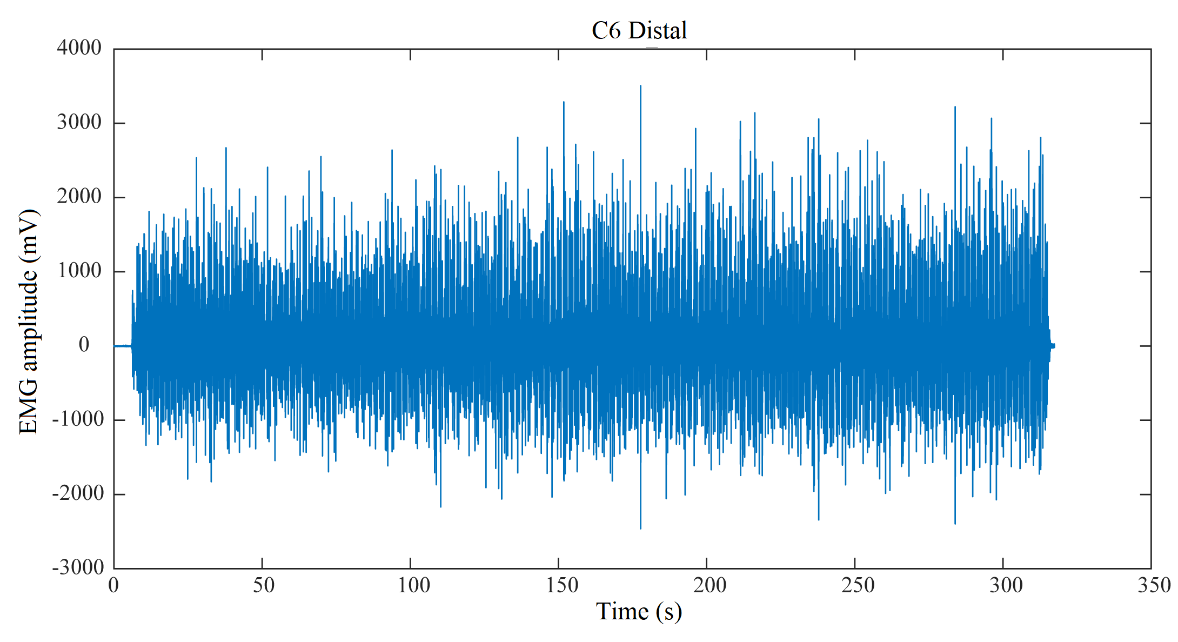

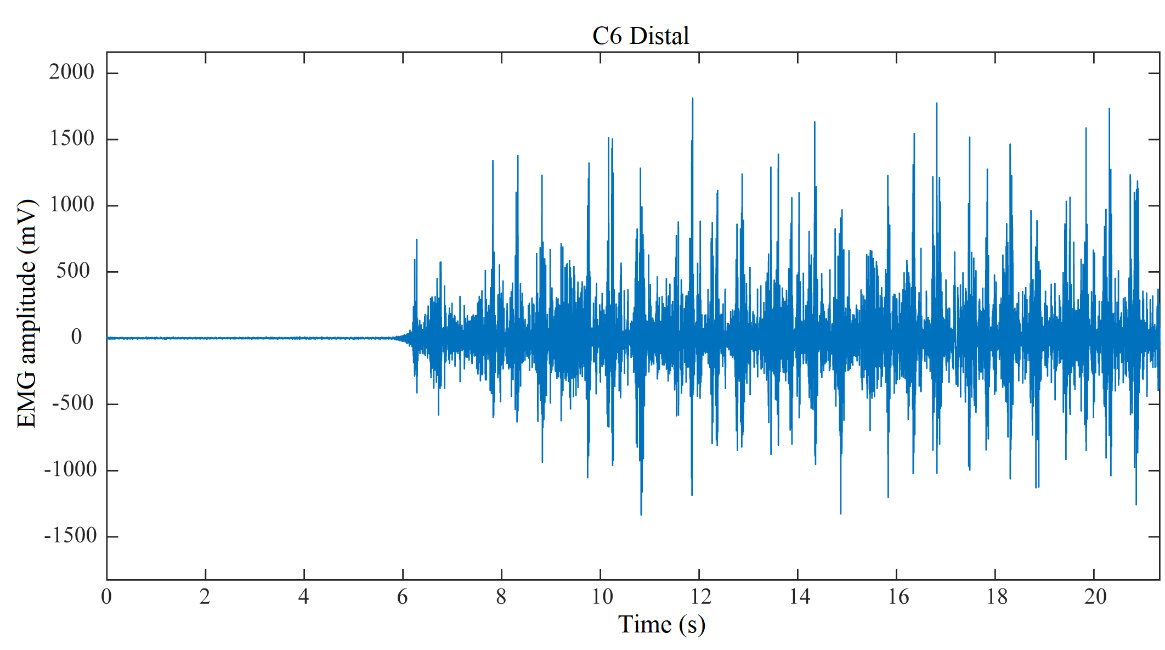


**G**


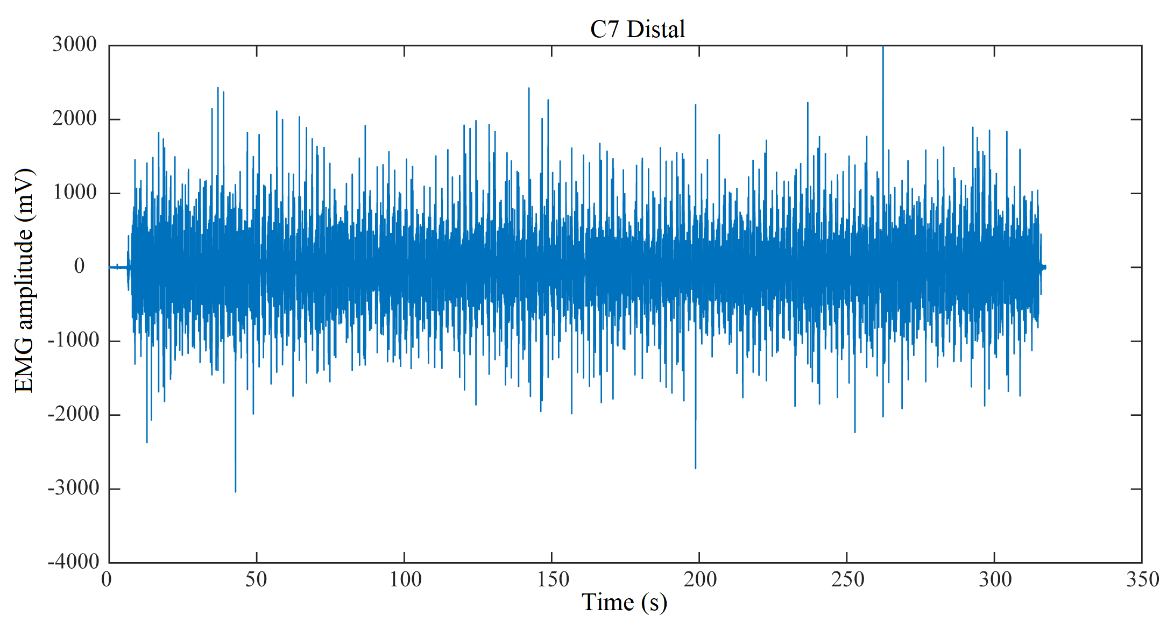

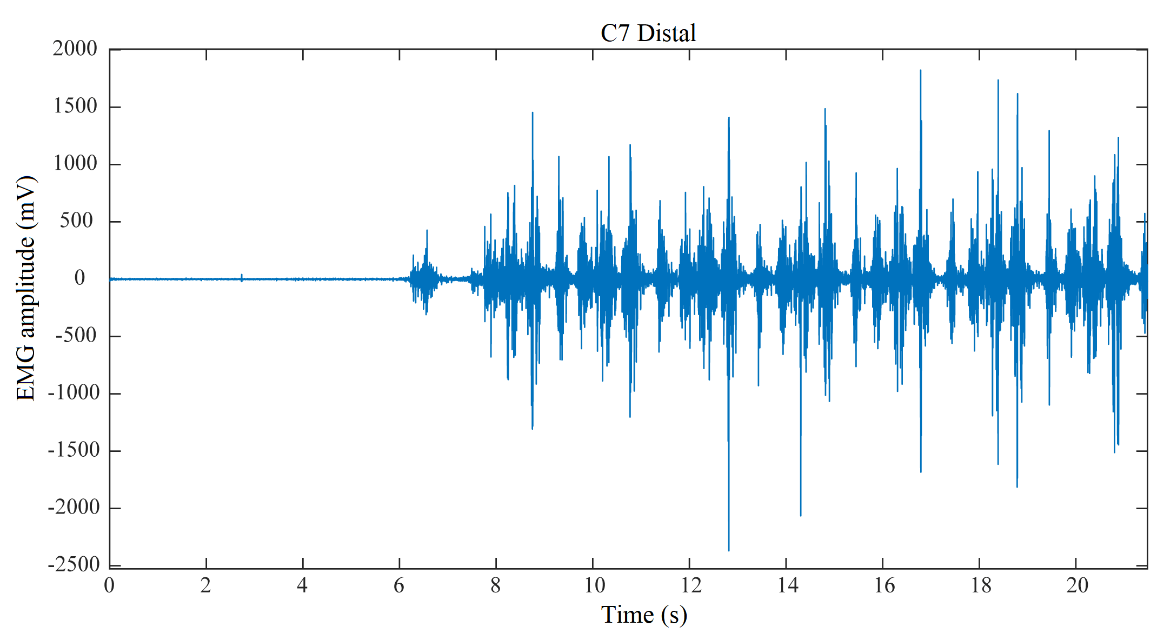


Figure 1: A-G) filtered EMG signal (superior part) and a zoom in on the baseline and the first seconds of piano playing (inferior part).

## A3. Baseline comparison between groups for piano performance variables

Table 1: Comparison between groups on piano performance parameters during the first 30-seconds for both Digital and Chord tasks.

|  | ***Digital task*** | | |  | ***Chord task*** | | |
| --- | --- | --- | --- | --- | --- | --- | --- |
|  | **ShortDuration** | **LongDuration** | **Statistical test** |  | **ShortDuration** | **LongDuration** | **Statistical test** |
|  | N=30; 9 ♀ | N=19; 10 ♀ |  |  | N=26; 11 ♀ | N=23; 8 ♀ |  |
| Incomplete cycles | 1.9 ± 2.0 | 1.7 ± 2.0 | t(47)=0.32; p=0.753 |  | 6.4 ± 4.5 | 5.9 ± 3.6 | t(47)=0.37; p=0.72 |
| Key velocity variance | 7.6 ± 4.9 | 7.3 ± 4.6 | T(782)=0.86; p=0.39 |  | 64.5 ± 141.7 | 50.6 ± 117.3 | T(243)=0.83; p=0.41 |
| Timing variance | 1.1e-4 ± 5.8e-5 | 1.0e-4 ± 5.5e-5 | t(47)=0.54; p=0.590 |  | 3.2e-4 ± 4.2e-4 | 3.5e-4 ± 4.4 e-4 | t(47)=-0.28; p=0.78 |

(α=0.05)

## A4. p-values of post-hoc analyses for main effect of time


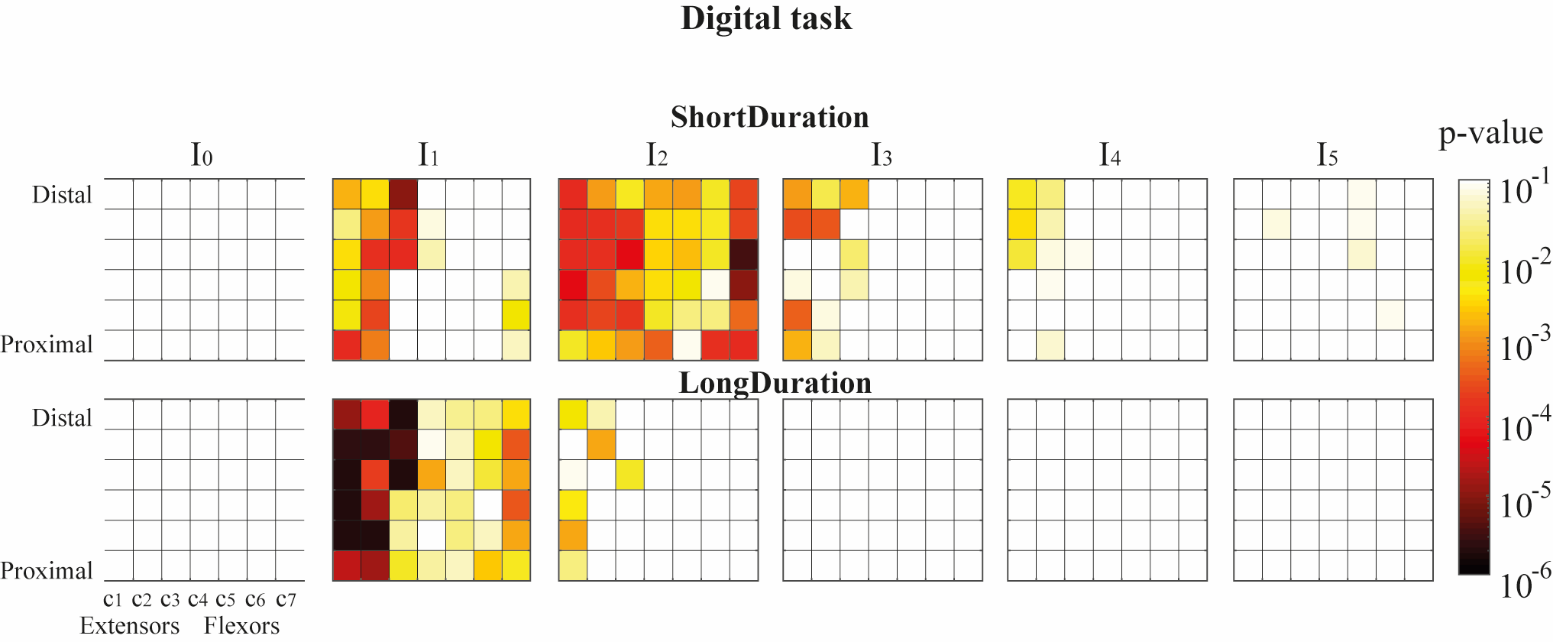


Figure 2: p-values of post-hoc analyses for main effect of time, for the *Digital* task. The c1 to c7 identifies the columns of electrodes as illustrated on Figure 8-B.


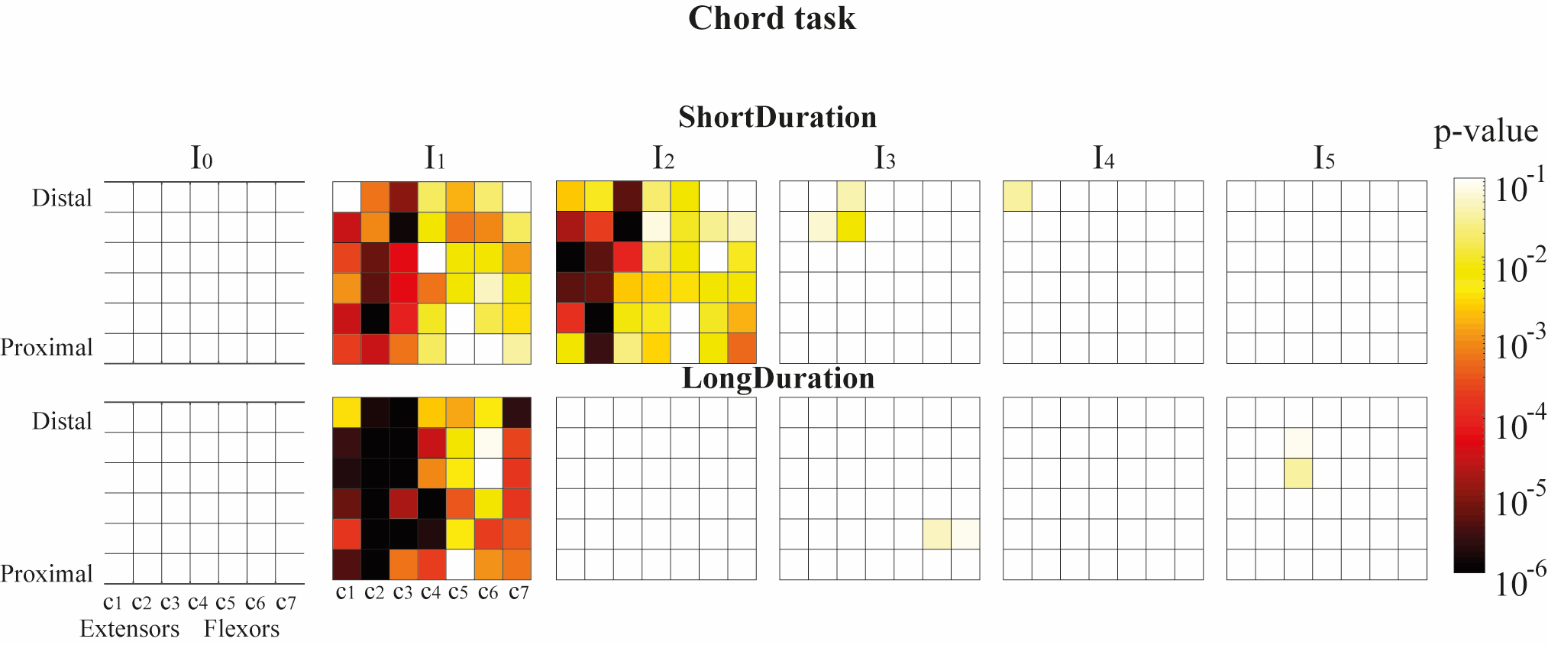


Figure 3: p-values of post-hoc analyses for main effect of time, for the *Chord* task. The c1 to c7 identifies the columns of electrodes as illustrated on Figure 8-B.
